# Light- and temperature-sensitive seizures are regulated by spatially distinct cortex glial populations in the central nervous system

**DOI:** 10.1073/pnas.2605750123

**Published:** 2026-07-21

**Authors:** Govind Kunduri, Tanja Angela Godenschwege, Katherine Sankey, Kandahalli Venkataranganayaka Abhilasha, Usha Acharya, Jairaj K. Acharya

**Affiliations:** ^a^Center for Cancer Research, Cancer and Developmental Biology Laboratory, National Cancer Institute, Frederick, MD 21702; ^b^Biological Sciences Department, Florida Atlantic University, Jupiter, FL 33458

**Keywords:** glia, epilepsy, sphingolipids, glial heterogeneity

## Abstract

Epilepsy is characterized by recurrent seizures due to abnormal neuronal activity originating from a population of neurons. Seizures are often associated with abnormal glial cell function at the seizure focus. Studies have shown that each glial type, such as astrocytes, displays a significant degree of heterogeneity in their development, molecular signatures, and function depending on the brain region in which they are located. It is unknown, however, if such heterogeneity differentially influences or causes seizures. Previous studies in *Drosophila* have shown that aberrant Cortex glia (CG) function led to light-inducible (LI) seizures in Ceramide phosphoethanolamine synthase (*cpes*) and temperature-sensitive (TS) seizures in zydeco (*zyd^1^*) mutants. Here, we have optimized Gal4/Split-Gal4/Gal80/LexA drivers to specifically express a gene of interest throughout development in CG subpopulations in different parts of the brain including optic lobe (OL), central brain (CB), and ventral nerve cord (VNC). Using these tools, we performed brain region–specific CG rescue experiments in *cpes* and *zyd^1^* mutants. We found that OL- and CB-specific, but not VNC-specific CG expression of CPES significantly suppressed LI seizures in *cpes* mutants. In contrast, VNC- but not OL- or CB-specific CG expression of ZYD suppressed TS seizures. In a third model, expression and activation of transient receptor potential, dTRPA1, exclusively in the VNC-specific CG was sufficient to induce TS seizures in wild-type flies. Our findings suggest that regionally specialized CG subtypes differentially regulate seizure susceptibility in seizure models.

## Significance

Glia are present throughout the mammalian brain. Whether morphologically similar glia in different parts of the brain regulate neuronal function in a spatially circumscribed manner is an area of intense research. *Drosophila* cortex glia (CG) share some similarities with mammalian astrocytes, particularly in their ability to interact with neuronal soma. In this study, we developed intersectional genetic methods to restrict gene expression specifically to CG located in different parts of the brain. Using epilepsy models, we show that light- and temperature-sensitive (TS) seizures are differentially regulated by spatially distinct CG subpopulations. Central brain–specific CG are important for regulating light-inducible seizures, whereas ventral nerve cord–specific CG are essential for suppressing TS seizures.

Glia and neurons are the two major cellular components of the nervous system in both vertebrates and invertebrates. Although glial cells were first discovered in 1854, most insights into their roles in neuronal differentiation, organization, function, and survival have emerged only in the past two to three decades (1, 2). Recent studies emphasize that glia are equal partners in central nervous system (CNS) function rather than passive support cells (2–5). Accordingly, CNS pathologies are increasingly viewed as arising from primary defects in neurons or glia, rather than neurons alone (6). A key example of this shift is epilepsy research (7).

Epilepsy is a CNS disorder characterized by recurrent seizures caused by high-frequency action potentials and hypersynchronization of neuronal populations that affects over 65 million people worldwide (8, 9). Seizure development is often associated with complex structural and functional changes in astrocytes (reactive astrogliosis) within seizure foci following CNS insults (9). Once viewed as a secondary response to neuronal dysfunction, astrogliosis emerged as a direct driver of epilepsy, as exemplified by astrocyte-derived glial fibrillary acidic protein (GFAP) variants in Alexander disease (10–13). Although antiseizure drugs are effective in about 70% of patients, 30% remain drug-resistant, underscoring the need to investigate non-neuronal mechanisms involving glial cells (14–16). Astrocytes modulate neuronal activity and exhibit significant structural and molecular heterogeneity across brain regions in their development, molecular profiles, and physiology (17–20). The functional significance of this heterogeneity remains poorly understood, though it may optimize local neuronal circuit function (18, 19). Understanding regional glial specialization and dysfunction in epilepsy is therefore a critical unmet need for developing targeted therapies (9).

Presence of glial heterogeneity within the same class, across brain regions have also been indicated in a genetically tractable model organism *Drosophila melanogaster* (21–23). However, the functional significance of such regional heterogeneity remains unknown. In *Drosophila* CNS, Cortex glia (CG) envelop up to 100 neuronal cell bodies in a honeycomb-like structure and play a vital role in regulating neuronal hyperexcitability (21, 24–29). Previously, we have shown that Ceramide phosphoethanolamine synthase (CPES) plays an important role in membrane expansion, and absence of CPES leads to defective CG plasma membrane and development of light-inducible (LI) seizures (27, 30). We have shown that altered glial plasma membrane structure, specifically a failure to establish detergent resistant membranes (DRMs) is responsible for CG encapsulation defect in *cpes* mutants. It has been shown that loss of function of CG-specific Na^+^/Ca^+^, K^+^ exchanger (*Zyd*eco or NCKX), one of the first identified glial-specific genes involved in epileptic phenotypes, results in Temperature-sensitive (TS) seizures. Zydeco (ZYD) has been shown to influence glial Ca^2+^ signaling via regulation of microdomain Ca^2+^ oscillations at the plasma membrane. The *zyd^1^* mutant CG shows higher intracellular Ca^2+^ levels (31). Further, it has been shown that increased CG Ca^2+^ levels cause hyperactivation of calcineurin-dependent endocytosis of K2P channel, Sandman, resulting in the reduced ability of glial cells to buffer K^+^ around neuronal cell bodies resulting in increased neuronal excitability (29).

In this study, we optimized methods to express a gene of interest in CG subpopulations in different parts of the brain including optic lobe (OL), central brain (CB), and ventral nerve cord (VNC). Subsequently, using these tools and CG subpopulation–specific rescue experiments, we show that OL- and CB-specific CG are important in regulating LI seizures in *cpes* mutants. In contrast, VNC-specific CG plays an important role in regulating TS seizures in *zyd^1^* mutants. Further, we show that Ca^2+^ influx into VNC-specific CG was sufficient to induce TS seizures in wild-type (WT) flies. Together our results suggest that CG subpopulations across brain regions differentially regulate seizure susceptibility in different seizure models.

## Results

### Optimization of Gene Expression in CG Subpopulations Located in OL, CB, and VNC.

To express genes of interest in subsets of CG populations across brain regions, we utilized Gal4 and split-Gal4 lines generated by the Janelia Fly Light project (32–34). Gal4 driver lines that label CG in different brain regions, including OL, CB, and VNC have been described before (22). It was suggested that R65B12-Gal4, R9F07-Gal4, and R54D10-Gal4 labeled the CG of OL, CB, and VNC respectively in the adult brain (22). However, their developmental expression patterns were unknown and R54D10 and R9F07-based transgenes were not available from stock centers. Therefore, we regenerated R54D10-Gal4.DBD, R54D10-Gal80, R54D10-nlsLexAp65 (AD), R9F07-Gal4.DBD, and R9F07-Gal80 as described in *SI Appendix*. We combined these lines with published Gal4/SplitGal4 lines to promote or restrict gene expression in selected brain regions. We also screened Janelia FlyLight Gal4, split Gal4, LexA and Vienna tile (VT) collections to identify additional lines that likely express in CG of adult CNS (*SI Appendix*, Table S1). We used split-Gal4 system because single-enhancer Gal4 lines often show broad expression patterns, whereas split-Gal4 utilizes two different enhancers to restrict gene expression to cells where both enhancers are active to produce DNA-binding domain (DBD) and an activation domain (AD) (32, 35).

**Fig. 1.**
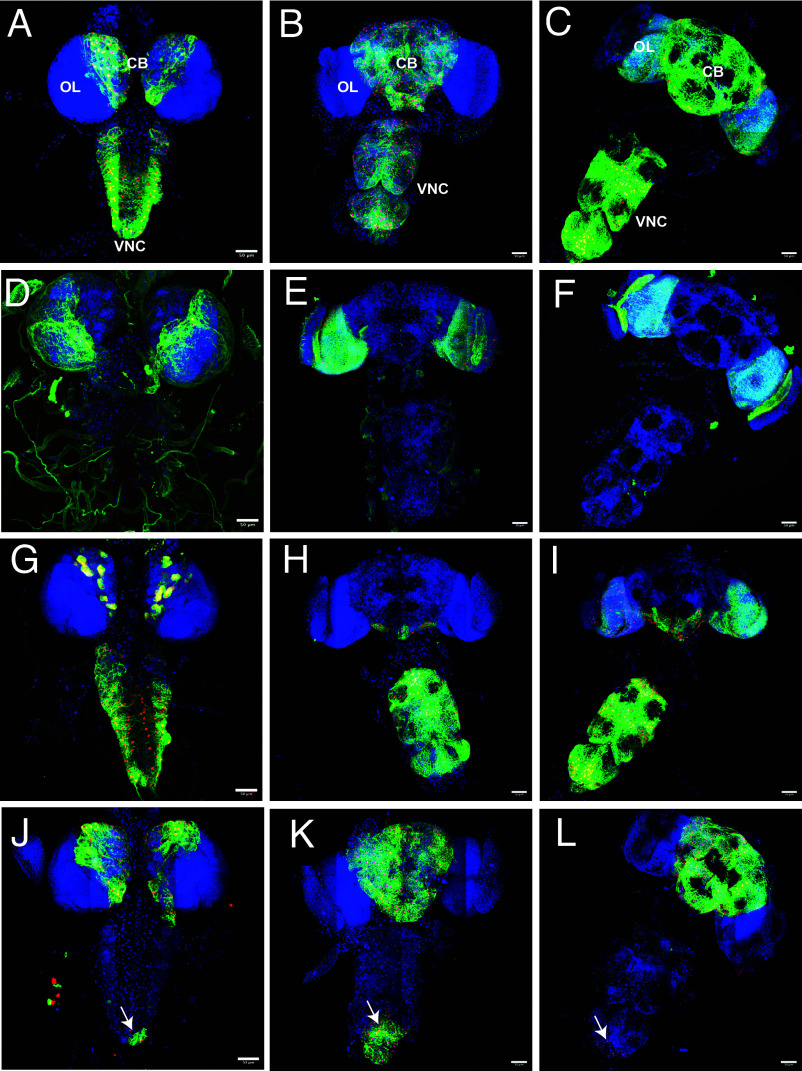
Labeling region-specific CG cells in *Drosophila* CNS. (*A*–*C*) Expression pattern of CB+VNC1-Gal4 in fly CNS, during 3rd instar larval stage (*A*), 48 h postpupation (*B*), and 7-d-old adult (*C*). (*D*–*F*) Expression pattern of OL1-Gal4 in fly CNS during 3rd instar (*D*), 48 h postpupation (*E*), and 7-d-old adult (*F*). (*G*–*I*) Expression pattern of VNC2-Gal4 during 3rd instar (*G*), 48 h postpupation (*H*), and 7-d-old fly CNS (*I*). (*J*-*L*) Expression pattern CB1-Gal4, during 3rd instar (*J*), 48 h postpupation (*K*), and 7-d-old fly CNS (*L*). Arrows indicate continued expression of markers at the tip of VNC. Green (UAS mCD8GFP), red (mCherryNLS), and blue (DAPI). (Scale bar, 50 μm.)

Nrv2-Gal4 specifically labels all CG subtypes and ensheathing glia in the adult brain. To visualize Gal4/split-Gal4 expression, we used Upstream activating sequence (UAS) mCD8GFP (to mark cell membranes) and UAS-mCherryNLS (to label nuclei) reporters (36). Our FlyLight screen identified VT038983 as a candidate that strongly expresses in CG of adult CB and VNC but weakly in OL (*SI Appendix*, Table S2). Combining Nrv2-p65(AD) (21) with VT038983-Gal4.DBD generated CB+VNC1-Gal4 which specifically labeled CB- and VNC-specific CG in third-instar larvae, pupae (Fig. 1 *A* and *B*). In adults, however, expression occurred in OL resulting in more generic CG expression pattern (Fig. 1*C* and *SI Appendix*, Table S2). A second combination R9F07-Gal4.DBD with VT038983-p65(AD) (CB+VNC2-Gal4) showed a similar pattern, except adult OL where one of the two OLs had weaker expression (*SI Appendix*, Fig. S1 *A*–*C*).

R65B12-Gal4 labels OL-specific CG of adult brain (22). Developmental analysis showed that in addition to OL-specific expression, it also expressed in neurons of the 3rd instar brain (*SI Appendix*, Fig. S1*D*) and in CG of the CB and VNC during pupal stages (*SI Appendix*, Fig. S1*E*). In adults, expression was restricted mainly to OL-specific CG, and weakly in neurons (*SI Appendix*, Fig. S1*F*). Because no alternative OL-specific CG drivers available, we optimized R65B12-Gal4 using Gal80 repression. Coexpression with pan-neuronal nsyb-Gal80 suppressed neuronal expression, while R9F07-Gal80 blocked the expression in CG of CB and VNC during pupal stages without affecting OL expression. This combination termed OL1-Gal4, restricted expression to OL-specific CG throughout development (Fig. 1 *D*–*F* and *SI Appendix*, Table S2).

R54D10-Gal4 labels CG in the adult VNC and subesophageal ganglion (SEG) (22). To assess developmental expression, we combined Nrv2-p65(AD) with the R54D10-DBD to generate VNC1-Gal4. The expression of this Gal4 was primarily restricted to VNC in 3rd instar and pupal brains (*SI Appendix*, Fig. S2 *A* and *B*), but in adults expression also appeared in SEG and OL, in addition to VNC (*SI Appendix*, Fig. S2*C* and Table S2). Overall, consistent with previous reports, R54D10 expression was absent from most of the CB at all stages. Similar results were obtained with VT038983-p65(AD), and R54D10-Gal4.DBD (VNC2-Gal4; Fig. 1 *G*–*I*), although some CG expression in CB neuroblast colonies in third-instar brains, which disappeared in pupae and adults (Fig. 1 *G*–*I*). Together, these results suggest that R54D10 expression is mostly excluded in the CB and the expression is primarily restricted to VNC, SEG, and OL.

Because no Gal4/split-Gal4 drivers are currently available to label CG of the CB, we combined split-Gal4 system with the LexA Killer zipper (KZip+) system. In this approach, a dominant-negative repressor (Gal4DBD/KZip+) is expressed from a third promoter, to suppress split-Gal4 activity in selected cells (37). Since CB+VNC1-Gal4 labels CG broadly in the adult brain (Fig. 1 *A*–*C*) we used R54D10-nlsLexAp65AD to repress expression in VNC, SEG, and OL of adult brain (Fig. 1 *G*–*I* and *SI Appendix*, Fig. S2 *A*–*C*). R54D10-nlsLexAp65AD activates 13x LexAop2-KZip+ 3xHA in these regions, leaving expression only to CB (*SI Appendix*, Fig. S3 *D*–*F*), In adult brains, the gene expression was consistently restricted primarily to CB (Fig. 1*L*), although repression was not complete at the VNC tip in third-instar, and pupal brains, likely due to insufficient KZip+ 3xHA expression (Fig. 1 *J* and *K*). Adult brains however showed a stronger repression, as a result only a weak expression was visible at the tip of VNC (Fig. 1 *J*–*L*, CB1-Gal4). Similar results were obtained using Nrv2-p65(AD) with Wrapper-Gal4.DBD (Ctx-Gal4, *SI Appendix*, Fig. S3 *A*–*C* and *G*–*I*; CB2-Gal4). Taken together R54D10-nlsLexAp65 > 13xLexAop2KZip+3xHA effectively represses split-Gal4 expression in most parts of the VNC and OL CG, leaving expression largely restricted to the CB (*SI Appendix*, Table S2).

### CB–Specific CG Is Important in LI Seizures.

**Fig. 2.**
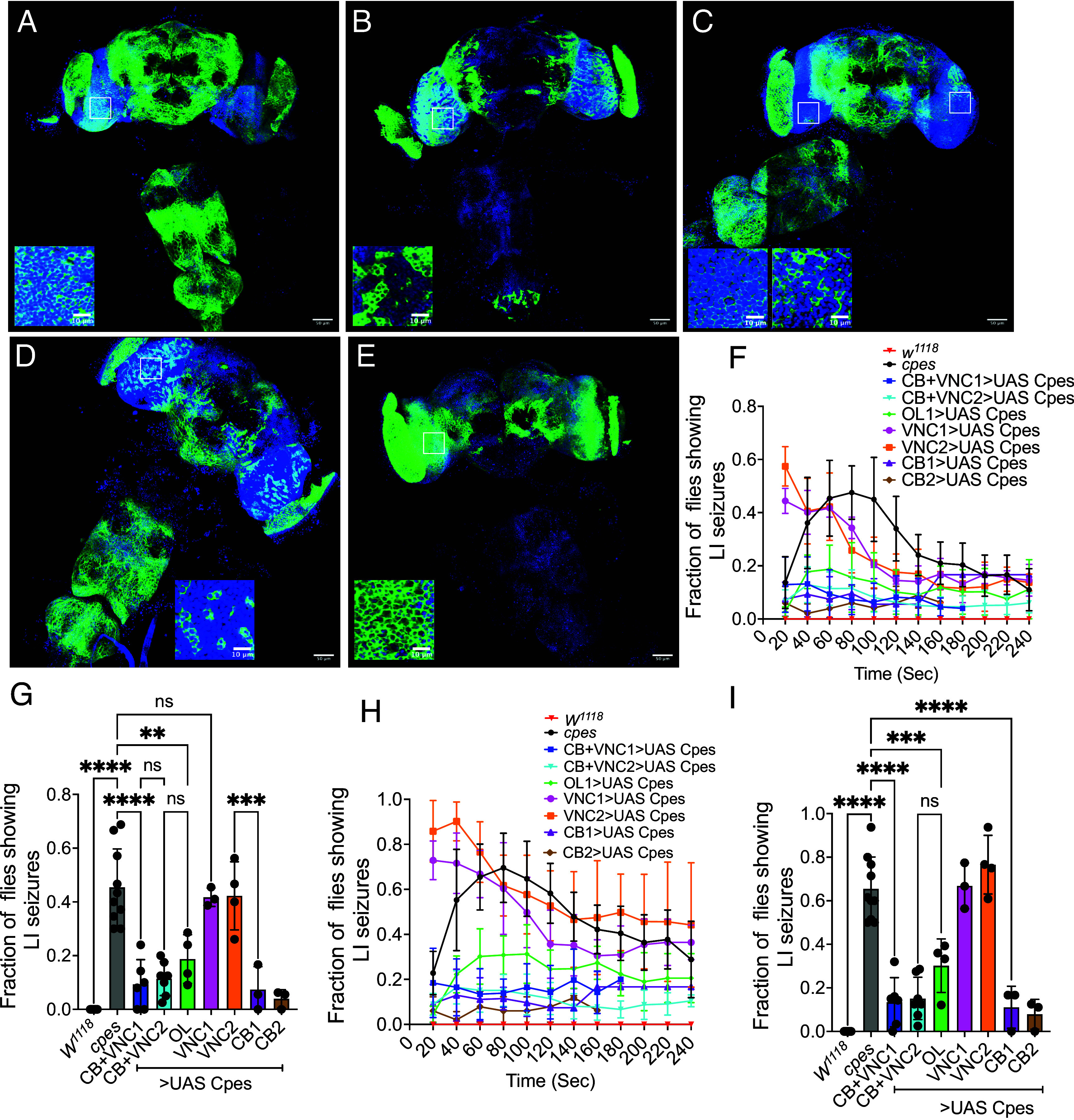
Evaluation of defective CG and LS seizure rescue with region-specific Gal4 drivers in *cpes* mutants. (*A*–*E*) Adult CNS, wherein CG is labeled with Wrapper-nlsLexA-p65 and LexAop2-rCD2-GFP, in various genetic backgrounds including in WT (*A*); *cpes* mutant with UAS-Cpes alone (*B*); *cpes* mutant background with UAS-Cpes expressed via CB+VNC1-Gal4 (*C*); *cpes* mutant background with UAS-Cpes expressed in the VNC via VNC2-Gal4 (*D*). *cpes* mutant background with UAS-Cpes expressed in the OLs with the combination of OL1-Gal4 (*E*); the inset shows a small portion of OL-specific CG in all backgrounds (*A*–*E*). (*F* and *G*) Quantification of fraction of flies showing complete seizures as a function of time. (*G*) Statistical analysis for the complete LI seizures at time point 1 min/60 s. (*H* and *I*) Quantification of fraction of flies showing complete and partial seizures. (*I*) Statistical analysis for the complete and partial LI seizures phenotype at time point of 1 min/60 s. Each data point in *F* and *H* represents an average fraction of seizure behavior from a minimum of 50 flies from three independent experiments. Each dot in *G* and *I* represents a fraction of seizure behavior in each independent experiment consisting of 15 to 30 flies. Green (UAS-mCD8GFP), Red (mCherryNLS), Blue (DAPI). [Scale bar, 50 μm (*A*–*E*) and 10 μm (*Inset*).] The 2way ANOVA multiple comparison was used to calculate *P* values where ****P ≤ 0.0001; ***P ≤ 0.001; **P ≤ 0.01; *P ≤ 0.05 and ns *P* > 0.05.

Having largely optimized Gal4/split Gal4 drivers for region-specific CG expression across developmental and adult stages, we investigated whether CG in different brain regions differentially regulate seizures in models of light- and temperature-inducible seizures. We previously showed that *cpes* null mutants display LI seizures (27). To identify brain regions that play an important role in LI seizures, we performed region-specific CG rescue experiments using optimized Gal4/SplitGal4/LexA drivers expressing UAS-Cpes. We hypothesized that expressing UAS-Cpes in a specific brain region would rescue the CG in that region. For instance, expression of UAS-Cpes in the VNC should rescue the CG only in the VNC but not in the OL or CB. Similarly, expression of UAS-Cpes in the OL or CB should rescue the CG only in the OL or CB respectively. To investigate, if this is the case, we first constitutively labeled CG membranes using generic CG driver Wrapper-nlsLexAp65AD and LexAop2-rCD2-GFP. In contrast to controls, *cpes* mutant brains showed CG defects throughout VNC, CB, and OL (Fig. 2 *A* and *B*). Expression of UAS-Cpes in CB- and VNC-specific CG using CB+VNC1-Gal4 fully restored the CG defects in respective brain regions (Fig. 2*C*). However, we observed only a partial rescue of the CG in OL (Fig. 2 *C*, *Left* vs. *Right* *Inset*). Interestingly, expression of UAS-Cpes in VNC-specific CG using VNC2-Gal4 restored CG integrity in VNC and SEG, but not in the CB and OL, consistent with its expression pattern during development (Fig. 1 *G*–*I* vs. Fig. 2*D*).

Although VNC2-Gal4 also drives weak expression in the adult OL (Fig. 1*I*), no rescue of CG was observed in this region (Fig. 2*D*), likely due to absence of its expression during development. This suggests that the presence of CPES protein during larval and pupal stages is essential for rescue of CG, and late expression in the adult brain may not restore CG that were already disrupted during development. Similarly, we found that expression of UAS-Cpes in OL-specific CG using OL1-Gal4 fully restored CG defects in OL, but not in the VNC region, consistent with the expression pattern (Fig. 2*E*). We also observed partial rescue in CB-specific CG, in addition to OL-specific rescue (Fig. 2*B* vs. Fig. 2*E*), probably due to insufficient repression of R65B12-Gal4 by R9F07-Gal80 during pupal stages (*SI Appendix*, Fig. S1 *D*–*F*).

Because CPES protein localizes to the trans-Golgi and plasma membrane under physiological conditions (27, 30, 38), we examined HA-tagged UAS-Cpes expression in *cpes* mutants using above-mentioned drivers. Adult fly brains were dissected, immunostained for HA-tag, and visualized using spinning disk confocal microscopy. When expressed using CB+VNC1-Gal4 (*SI Appendix*, Fig. S4*A*), CPES protein was detectable in adult CB, VNC, and OL consistent with its expression pattern (Fig. 1 *A*–*C*). Note that CG appears defective in OL, which is consistent with the absence of CPES-HA protein in this region during development. Similarly, expression in the VNC+SEG+OL using VNC1-Gal4 or VNC2-Gal4, resulted in detectable CPES-HA in all these respective regions. Notably, the CG in OL and CB appeared abnormal in the adult brain (*SI Appendix*, Fig. S4 *B* and *C*), consistent with the lack of CPES-HA expression in these regions during development (Fig. 1 *G*–*I*). When expressed in CB, CPES-HA was readily detectable in adult CB but not in OL or VNC (*SI Appendix*, Fig. S4*D*). It should be noted that the nuclear staining seen in VNC and OL (*SI Appendix*, Fig. S4*D*) was due to simultaneous expression of HA-tagged KZip+ repressor in these regions driven by R54D10LexAp65AD. Surprisingly, when expressed using OL1-Gal4, CPES-HA was not detectable in the adult brain (*SI Appendix*, Fig. S4*E*). To further evaluate its expression during development, we imaged immunostained CNS from third-instar, pupa, and adult stages. We found that CPES-HA was strongly expressed during pupal stage but not detectable during third-instar and adult stages (*SI Appendix*, Fig. S4 *F*–*H*). However, CG morphology was rescued in the adult brain (Fig. 2*E*), even though the protein was not detectable in the adult (*SI Appendix*, Fig. S4 *E* and *H*). In this case the discrepancy between mCD8GFP and UAS-Cpes-HA expression (Fig. 1 *D*–*F* vs. *SI Appendix*, Fig. S4 *F*–*H*) is likely due to excessive repression by R9F07Gal80 and differences in protein stability. Regardless of lack of CPES protein detection, the rescue of CG in the adult OL (Fig. 2*E*), is likely due to protein expression during the pupal stage, which appears to be sufficient to restore its morphology. Taken together these results suggest that brain subregion–specific CG rescue with UAS-Cpes mostly mirrors the expression pattern observed for the corresponding Gal4/split-Gal4 drivers with fluorescent reporters.

To further investigate how rescuing CG in specific brain subregions affects LI seizures, we expressed UAS-Cpes using one OL, two sets of CB, and two sets of VNC-specific Gal4/Split Gal4 drivers in *cpes* mutants. The *cpes* mutant adult flies (3 to 4 wk old) were sensitive to fluctuations in light intensity and showed robust seizures when shifted from dark to ambient light conditions (27). They typically showed two behavioral phenotypes upon shifting from dark to light: complete seizure/paralysis where flies fall to the bottom and lie on their backs or sides until recovery or partial seizure where flies fall to the bottom and remaining immobile while standing on their legs (Movie S1). However, we observed that an individual fly could show partial or complete or no seizure interchangeably in different assays (*SI Appendix*, Data S1). The adult CNS region–specific CG rescue flies were aged for 3 to 4 wk and tested for LI seizures. We first assayed for population-level analysis wherein 20 to 40 flies were taken in each vial, dark adapted overnight, and then exposed to light at the time of assay as described in *Materials and Methods*. At least 3 to 10 vials were analyzed for each genotype from independent experiments. Flies showing complete or partial or both seizure types were scored manually by assessing the videos at various time frames and plotted as line and bar diagram. In contrast to WT flies, *cpes* mutant flies showed a significant amount of LI seizures (complete seizures only—Fig. 2 *F* and *G*, and complete as well as partial seizure—Fig. 2 *H* and *I*). The statistical difference for these data was analyzed at the 60 s time point (Fig. 2 *G* and *I*). Rescue of Cpes expression in the CG of CB+VNC using two different split Gal4 drivers significantly suppressed LI seizures, suggesting the importance of this CG in LI seizures (Fig. 2 *G* and *I* and Movie S2). Interestingly, restoring Cpes expression just in the VNC and SEG using two different split-Gal4 drivers did not rescue LI seizures, suggesting CG in the VNC regions may not play a major role in suppression of LI seizures (Fig. 2 *G* and *I* and Movie S2). Contrary to VNC-specific rescue, expression of UAS-Cpes using two different CB-specific CG drivers significantly suppressed LI seizures (Fig. 2 *G* and *I* and Movie S2), suggesting an important role for CB-specific CG in LI seizures. We also observed a partial suppression of LI seizures with OL-specific CG (Fig. 2 *G* and *I* and Movie S2). It should be noted that as mentioned above, OL-specific expression of UAS-Cpes also partially rescues the CB-specific CG, suggesting this partial rescue may be due to rescue of CG in the CB. However, it is also possible that both OL and CB-specific CG contribute to suppression of LI seizures.

To further observe individual fly behaviors more closely we performed single fly analysis, wherein individual flies from each genotype were taken into separate vials and subjected to light-sensitive assay. In this assay we took around 20 flies for each genotype and their light sensitivity was manually scored and plotted as a line diagram. Consistent with the population-level analysis (Fig. 2 *F*–*I*), we found that CB- and OL-specific expression of UAS-Cpes significantly suppressed LI seizures, whereas no suppression was seen with VNC-specific expression (*SI Appendix*, Fig. S5 *A* and *B*). It should be noted that in population level analysis VNC+SEG-specific CG rescues showed increased sensitivity to light at earlier time points and subsequently showed faster recovery kinetics compared to *cpes* mutants (Fig. 2 *F* and *H* and Movie S2). However, such differences were not seen in the individual fly analysis, perhaps due to reduced sample size and large variations in time points at which an individual fly experiences seizures that ranged between 5 and 60 s. Taken together these results suggest that largely CB-specific CG and to some extent OL-specific CG play an important role in suppression of LI seizures in *cpes* mutants.

**Fig. 3.**
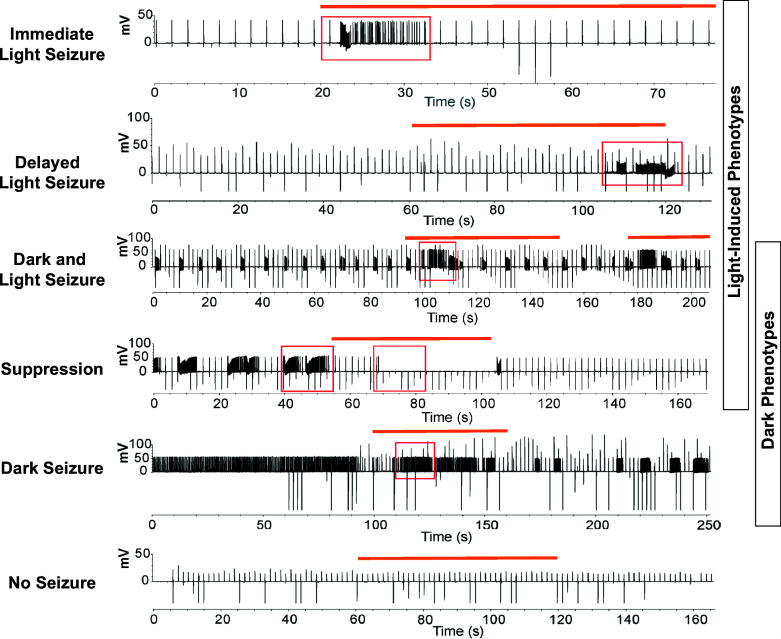
Electrophysiological recordings in cpes mutants confirm important role of CB in suppression of LI seizures. Distinct electrophysiological responses of DLMs in individual *cpes* mutants. Orange bars indicate exposure to light and rectangular boxes (red) indicate regions that are enlarged in the spike burst traces provided in *SI Appendix*, Fig. S6.

To measure fly seizure behavior more accurately and distinguish it from potentially intrinsic motor related abnormalities, electrophysiological recordings were obtained from *cpes* mutants and its rescue animals. Electrophysiological recordings from the dorsal longitudinal flight muscles (DLMs) are an established approach for assessing seizures in *Drosophila* (39–45). We used low-frequency giant fiber (GF) stimulation (0.5 Hz) to ensure recording electrodes are in place and to assess potential synaptic failures after seizure. The GF-DLM response latency was not significantly different between WT flies and *cpes* mutants at room temperature (*SI Appendix*, Fig. S5*C*), suggesting the function of the GF-DLM was not impacted in *cpes* mutants. We recorded activity in both DLMs in immobilized live animals first in darkness and subsequently in response to light stimulation. The flies exhibited several distinct phenotypes upon recording and about 14% of *cpes* mutant recordings had synaptic failures after seizure [Fig. 3 and *SI Appendix*, Fig. S6 (for expanded view of boxed regions in Fig. 3) and *SI Appendix*, Fig. S5*D*]. First, light triggered seizures with 1 to 3 bursts are seen in 45% of flies with two subtypes (immediate and delayed, Fig. 3 and *SI Appendix*, Fig. S5*D*). These bursts were usually short, not rhythmic and not persistent in the light. In several flies the LI seizures could be repeated after a 1 min dark period, other cases required longer resting periods (about 5 to 15 min). In addition, the seizures were typically synchronized in both DLMs with subtle differences and only in rare cases one-sided seizures (<3%) were observed. Second, about 30 to 40% of *cpes* mutant flies had seizures even before light stimulation (dark or ongoing seizures, Fig. 3*A* and *SI Appendix*, Fig. S5*D*). These seizures were either rhythmic or aberrant, and in some cases, light enhanced (dark and light seizures) or inhibited (suppression) the seizures including the 0.5 Hz GF-triggered responses. We termed it suppression as opposed to synaptic failure after seizure since the inhibition of the GF-triggered responses did correlate with end of a seizure and was also observed in the absence of any seizures in some animals. Finally, some of the mutants showed no phenotype in light or darkness (no seizures). In contrast, to the behavioral assays with freely moving animals, the electrophysiological assays had a wider variety of seizure phenotypes, which may be caused by various additional sensory stimuli and the stress animals were exposed to when recorded from. For example, the immobilization or insertion of electrodes into the flies, may have resulted in “sensory stress” in *cpes* mutants that was sufficient to trigger seizures. The underlying 0.5 Hz stimulation is unlikely to be the cause, as it has been shown to be insufficient to trigger seizures in WT or other types of seizure mutants (40–45). Furthermore, we confirmed with passive recordings, the presence of dark seizures before light onset in several animals. We have quantified the light-induced and dark phenotypes as well as the individual electrophysiological phenotypes of *cpes* mutants and its rescue animals in *SI Appendix*, Fig. S5 *D*–*F*. Due to the high variability of the electrophysiological phenotypes, we statistically compared the total number of flies with light-induced phenotypes between the mutants and rescue animals. Since light-induced phenotypes were also observed in the presence of dark seizures, and suppression similar to synaptic failure is likely to cause paralysis (40, 42, 45), we analyzed all light-induced phenotypes between genotypes (*SI Appendix*, Fig. S5*E*) separately from dark-specific phenotypes (*SI Appendix*, Fig. S5*F*). The CB-specific CG rescue showed significantly lower light-induced phenotypes compared to the VNC-specific CG rescue, suggesting CB-specific CG play an important role in suppression of LI seizures (*SI Appendix*, Fig. S5*E*). In contrast, dark phenotypes did not show a significant difference between CB- and VNC-specific CG rescues, but only a significant difference between CB+VNC rescue and VNC-specific rescue (*SI Appendix*, Fig. S5*F*). Taken together, the behavioral and electrophysiological data suggest that the CB- and OL-specific CG but not VNC-specific CG play a major role in suppression of LI seizures.

### VNC–Specific CG Is Important in TS Seizures in Zyd^1^ Mutants.

**Fig. 4.**
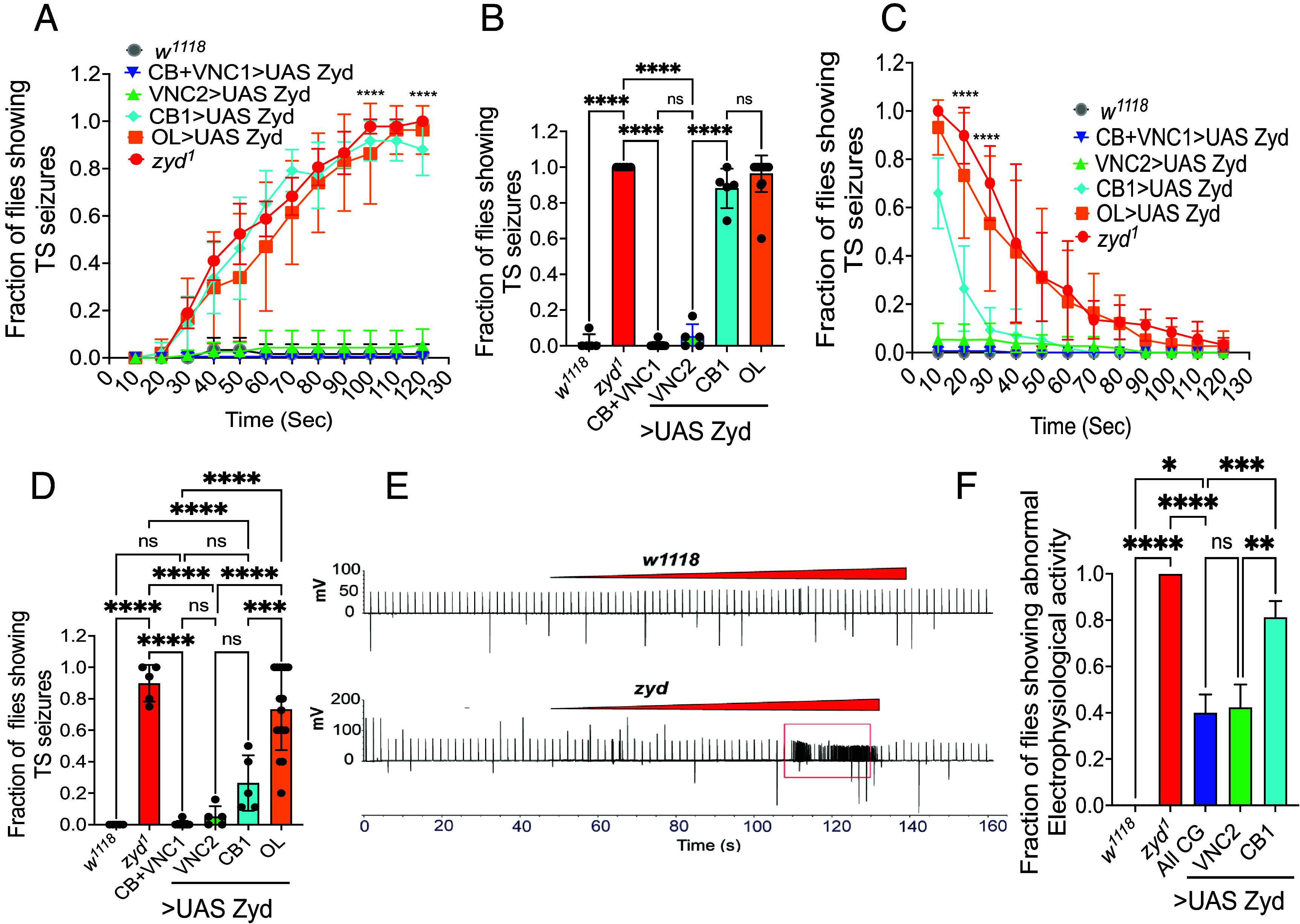
ZYD expression in the VNC-specific CG is sufficient for suppression of TS seizures in *zyd*^1^ mutants. (*A*) Measurement of TS seizures in *zyd^1^* mutants and various brain region–specific CG rescues and their effect on suppression of TS seizures as a function of time. UAS-Zyd was expressed using CB+VNC1-Gal4, VNC2-Gal4, CB1-Gal4, OL (R65B12Gal4). (*B*) statistical analysis of data in *A* at 120 s time point. (*C*) Measurement of seizure recovery in *zyd*^1^ mutants and brain region–specific CG rescues with UAS-Zyd expression as a function to time. (*D*) statistical analysis of seizure recovery of data in *C* at time points of 20 s. Each data point in *A* and *C* represents a minimum of 50 flies from five independent experiments. Each dot in *B* and *D* represent an independent experiment with 10 flies each. (*E*) Electrophysiological recordings for TS seizures in WT and *zyd^1^* mutants. Rise in temperature is indicated with a red scalene triangle. The red rectangular box indicates the region that is enlarged in the spike burst trace provided in *SI Appendix*, Fig. S7*C*. (*F*) Quantification of seizure behavior observed in electrophysiological recordings. At least 10 to 30 individual flies were analyzed for each genotype. The two way ANOVA multiple comparison was used to calculate *P* values where ****P ≤ 0.0001; ***P ≤ 0.001; **P ≤ 0.01; *P ≤ 0.05 and ns *P* > 0.05.

Hypomorphic zydeco (zyd) mutants show strong TS seizures (31). ZYD protein was shown to be expressed in all CG, but unlike *cpes* mutant, *zyd^1^* mutants do not show CG morphological defects, and acute CG-specific UAS-Zyd expression rescues TS seizures (31). Since CB- and OL-specific CG are involved in the regulation of LI seizures, we intended to determine whether these regions also influence TS seizures in *zyd^1^* mutants. Although *cpes* mutants also showed TS seizures, the phenotype is considerably weaker and transient in nature, with individual flies falling on their back and getting up within 5 to 10 s at high temperature (Movie S3). In contrast, *zyd^1^* mutants exhibited sustained seizures at high temperatures while remaining on their back or sides (Movie S4). The *zyd^1^* mutants (3 to 5 d old) developed complete seizures within 2 min at 38.5 °C and recovered within 3 min at room temperature; seizures could be reinduced in the same flies immediately after recovery. Under similar treatment conditions we did not observe significant TS seizures in WT flies. Analysis of motor phenotypes using negative geotaxis assay showed that *zyd^1^* and *cpes* mutants had significantly reduced locomotor activity compared to WT flies (*SI Appendix*, Fig. S7*D*). To investigate brain regions that are involved in TS seizures, we performed brain region–specific rescue experiments in *zyd^1^* mutants using UAS-Zyd and drivers optimized in Fig. 1. We crossed *zyd^1^*; +/+; UAS-Zyd female flies with various CG-specific Gal4/split-Gal4 driver males and collected male progeny for measurement of TS seizures. We have performed population and single-fly level assays on *zyd^1^* mutants and its rescues. For population assays, 10 to 20 male flies were collected per vial; for single-fly assays, one male per vial. All flies were aged for 5 d before being exposed to 38.5 °C in a water bath for the TS assay, as described previously (29, 31). Analyses of the population level assay showed that expression of UAS-Zyd in all CG completely rescued the TS seizures in *zyd^1^* mutants (Fig. 4 *A* and *B*). Interestingly, expression of UAS-Zyd in the VNC+SEG+OL alone was sufficient to rescue TS seizures in *zyd^1^* mutants as well (Movie S4). However, expression of UAS-Zyd in the CB or OL alone was not sufficient to rescue TS seizures in *zyd^1^* mutants (Fig. 4 *A* and *B* and Movie S4). These results indicate that VNC+SEG-specific CG play an important role in the suppression of TS seizures. Although CB- or OL-specific CG rescue did not suppress TS seizures, they did recover faster compared to *zyd^1^* mutants alone, suggesting an ancillary role for CG in these regions in seizure recovery (Fig. 4 *C* and *D*). Single fly behavioral analysis further confirmed these results wherein 70% of CB-specific rescue flies showed complete seizure by 50 s while the remaining 30% flies seized between 90 to 120 s. In contrast, VNC-specific CG rescue flies did not show significant seizures (*SI Appendix*, Fig. S7 *A* and *B*).

To evaluate seizure behavior more accurately and to distinguish from potentially motor related defects we used electrophysiological recordings. As before, we recorded from both DLMs seizures with an underlying 0.5 Hz GF stimulation (Fig. 4*E* and *SI Appendix*, Fig. S7*C*). We observed that left and right DLMs seizures were synchronized in all *zyd^1^* mutants and the respective rescue animals. We did not observe a significant difference in the GF-DLM response latency between WT and *zyd^1^* mutants when measured before the start of the assay at room temperature (*SI Appendix*, Fig. S5*C*). We obtained DLMs activity baseline recordings at room temperature for 30 to 60s, and subsequently gradually increased the temperature with a heat gun while monitoring the air temperature around the animals with a digital thermometer. In contrast to WT flies, all *zyd^1^* mutants showed seizure activity at temperatures 35 to 39 °C (Fig. 4 *E* and *F* and *SI Appendix*, Fig. S7*C*) and 44% of *zyd^1^* mutants exhibited synaptic failures after seizure. Interestingly, unlike in behavioral assays, all CG rescue flies also showed seizures with significantly lower frequency than *zyd^1^* mutants. Further, seizures in VNC-specific CG rescue flies were comparable to that of all CG rescue animals. However, significantly more CB-specific rescue flies showed seizure activity in comparison to VNC-specific rescue flies (Fig. 4*F*). Measurement of temperature onset of seizures among flies with seizures, showed no significant difference between all rescues, although a slight improvement was seen in CB-specific rescue relative to *zyd^1^* mutants (*SI Appendix*, Fig. S7*E*). It should be noted that this analysis does not factor in the animals that did not have seizures at higher temperatures. Taken together these results suggest that ZYD protein function in VNC is important for suppressing TS seizures in these mutants.

**Fig. 5.**
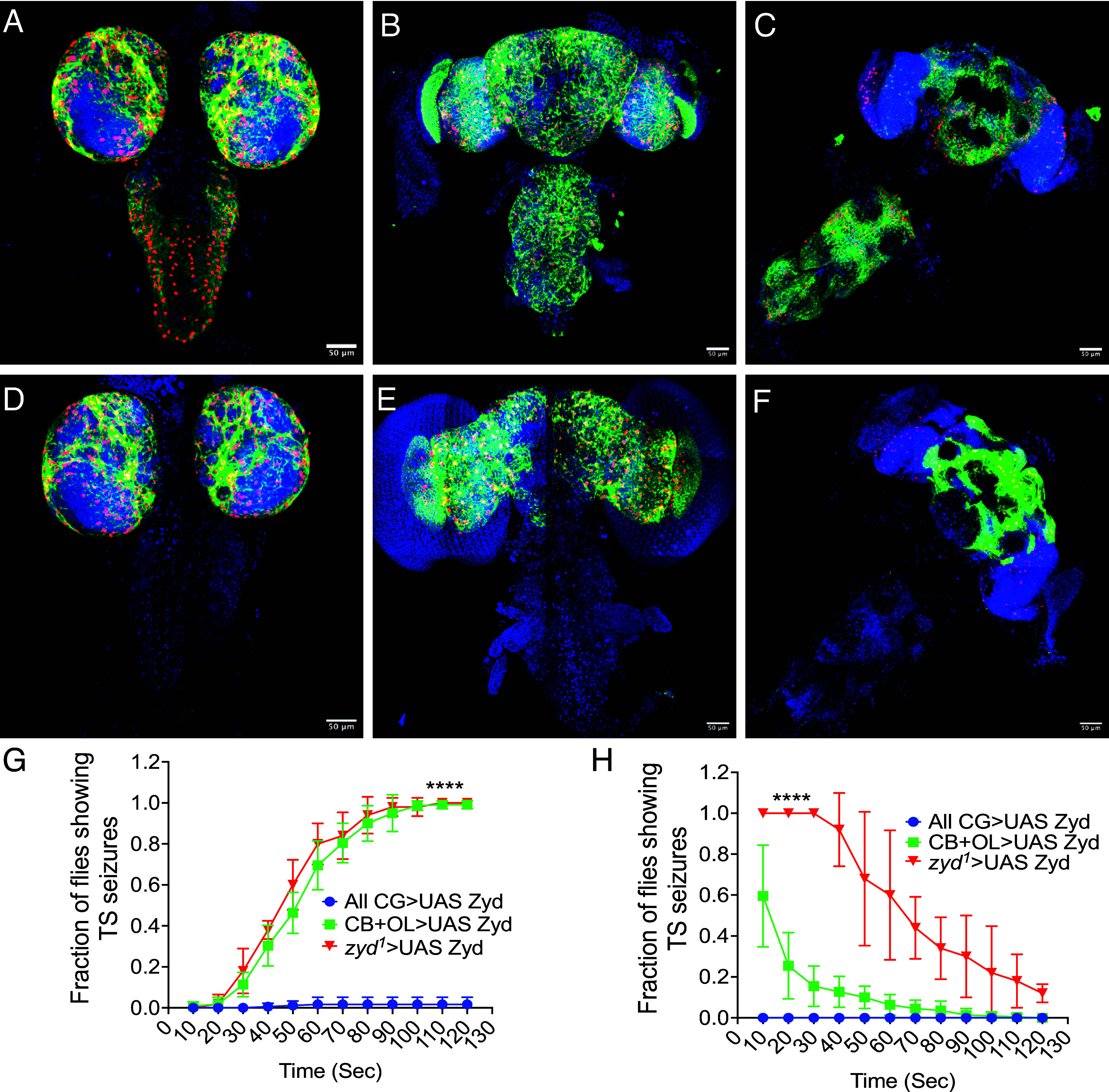
Zyd expression in the VNC-specific CG is essential for suppression of TS seizures in *zyd^1^* mutants. (*A*–*C*) Expression pattern of Wrapper-Gal4 in all CG (R54H02-Gal4 > mCD8GFP, mCherryNLS) in 3rd instar brain (*A*), 48 h postpupation (*B*), and 7-d-old adult CNS. (*D*–*F*) Expression pattern of R54H02-Gal4 after repression with R54D10-Gal80 > mCD8GFP, mCherryNLS in 3rd instar brain (*D*), 48 h post–pupation CNS (*E*), and 7-d-old adult CNS (*F*). [Scale bar, 50 μm (*A*–*F*).] (*G*) Quantification of TS seizures in all CG rescue and subsequent repression of UAS-Zyd expression in VNC alone using R54D10-Gal80 (CB+OL). (*H*) Quantification of heat-induced seizure recovery of flies in *G*, as a function of time. Each data point in *G* and *H* represents an average fraction of seizure behavior from about 100 flies from a minimum of five independent experiments.

To further investigate the importance of VNC-specific CG in suppression of TS seizures in *zyd^1^* mutants, we utilized the Gal80 system to repress UAS-Zyd expression in VNC (Fig. 5). Previous studies have shown that Wrapper-Gal4 specifically labels CG in all brain regions throughout development (Fig. 5 *A*–*C*) (22). To repress Wrapper-Gal4 expression in VNC, we coexpressed it with R54D10-Gal80. Consistent with its expression pattern (Fig. 1 *G*–*I* and *SI Appendix*, Fig. S2 *A*–*C*), R54D10-Gal80 repressed Wrapper-Gal4 expression in VNC, SEG, and OL of the adult brain, thereby limiting membrane mCD8GFP expression largely to CB (Fig. 5 *D*–*F*). However, it seems that R54D10-Gal80 repression is not complete in the OL of the adult brain as evidenced by weak detection of mCherryNLS (Fig. 5*F*). Wrapper-Gal4 mediated expression of UAS-Zyd completely rescued the TS seizures in *zyd^1^* mutants (Fig. 5*G*). Interestingly, repression of Wrapper-Gal4 mediated UAS-Zyd expression in the VNC using R54D10-Gal80 triggered TS seizures (Fig. 5*G* and Movie S5), suggesting the importance of Zyd in VNC for seizure suppression. Recovery analysis in this genetic background showed that UAS-Zyd expressed in CB+OL showed faster recovery compared to *zyd^1^* mutants alone suggesting some role of Zyd in these regions in seizure recovery (Fig. 5*H*). Single fly analysis further supported these results (*SI Appendix*, Fig. S8 *A* and *B*).

To evaluate whether CG subtype–specific Gal4 drivers express ZYD protein in a pattern similar to that of the mCD8GFP (Fig. 1), we generated C-terminal HA-tagged UAS-Zyd flies (UAS-Zyd-HA). We overexpressed UAS-Zyd-HA in CG subtypes in *zyd^1^* mutant background, and the dissected adult CNS was immunostained for the HA-tag and imaged. As previously shown, ZYD protein localizes to the CG plasma membrane (31); consistently, ZYD-HA was also localized to plasma membranes when expressed in all CG (*SI Appendix*, Fig. S8*C*). Further, when expressed in CB using two distinct drivers, consistent with mCD8GFP expression, ZYD-HA expression was restricted to the CB region (*SI Appendix*, Fig. S8 *D* and *E*). The punctate-like staining in VNC and OL corresponded to costaining of nuclear localized HA-tagged Killer Zipper (*SI Appendix*, Fig. S8*E*). When expressed in VNC, ZYD-HA expression was clearly detected in VNC+SEG consistent with that of VNC+SEG+OL-specific CG driver expression, although ZYD expression in OL was either weak or not detectable above the background level (*SI Appendix*, Fig. S8*F*). Surprisingly, ZYD-HA protein was not detectable in adult brains when expressed using the OL1-Gal4 (*SI Appendix*, Fig. S9*C*), despite being strongly expressed in pupal stage (*SI Appendix*, Fig. S9 *A* and *B*). This is likely due to excessive repression of R65B12Gal4 expression by R9F07 Gal80. Indeed, removing R9F07Gal80 restored R65B12Gal4 expression in the adult OLs (*SI Appendix*, Fig. S9*F*). However, lack of R9F07Gal80 resulted in leaky expression in CB and VNC during pupal stage that did not persist in the adult stage (*SI Appendix*, Fig. S9 *D*–*F*). Nevertheless, expression of UAS-Zyd using R65B12Gal4 alone did not rescue the TS seizures in *zyd^1^* mutants (Fig. 4 *A*–*D*), suggesting OL-specific expression of ZYD is not sufficient to suppress TS seizures in *zyd^1^* mutants.

Since we have measured complete paralysis at high temperature as a count of seizure, it is possible that VNC-specific CG rescue allows flies to recover sufficiently to stand, even if they are still experiencing seizures. To test this possibility, we performed single-fly walking experiments with *zyd^1^* and its rescue flies at high temperature. WT flies continued to walk throughout the assay duration at high temperature but *zyd^1^* mutants stopped walking within 80 s (*SI Appendix*, Fig. S10 *A* and *B*). As expected, expression of UAS-Zyd in all CG completely rescued seizures and therefore walking behavior (*SI Appendix*, Fig. S10*C*). Interestingly, most of the VNC-specific CG rescue flies continued to walk throughout the assay period (*SI Appendix*, Fig. S10*E*), whereas most of the CB-specific CG rescue flies stopped walking after 80 s due to seizures at high temperature (*SI Appendix*, Fig. S10 *D* and *F*). Walking tracks for individual flies for WT, mutant, and all the CG subtype–specific rescue flies were analyzed (*SI Appendix*, Fig. S10 *A*–*F* and Movie S6). Quantification of walking activity for the first 60 s showed a significant reduction in *zyd^1^* mutants compared to WT flies (*SI Appendix*, Fig. S10*G*). However, all other CG rescue flies showed higher or comparable activity to that of WT flies. Quantification of walking activity for the last 60 s, however, revealed that VNC-specific CG rescue flies have significantly higher activity compared to *zyd^1^* mutant and CB-specific CG rescue flies (*SI Appendix*, Fig. S10*G*). Although overall activity of VNC-specific CG rescue remained significantly lower than that of WT flies.

Taken together these results suggest that ZYD protein function in VNC- but not in CB-specific CG is crucial for suppression of TS seizures in *zyd^1^* mutants.

### Acute Ca^2+^ Influx in VNC-Specific CG Via dTRPA1 Activation Triggers TS Seizures in WT Flies.

**Fig. 6.**
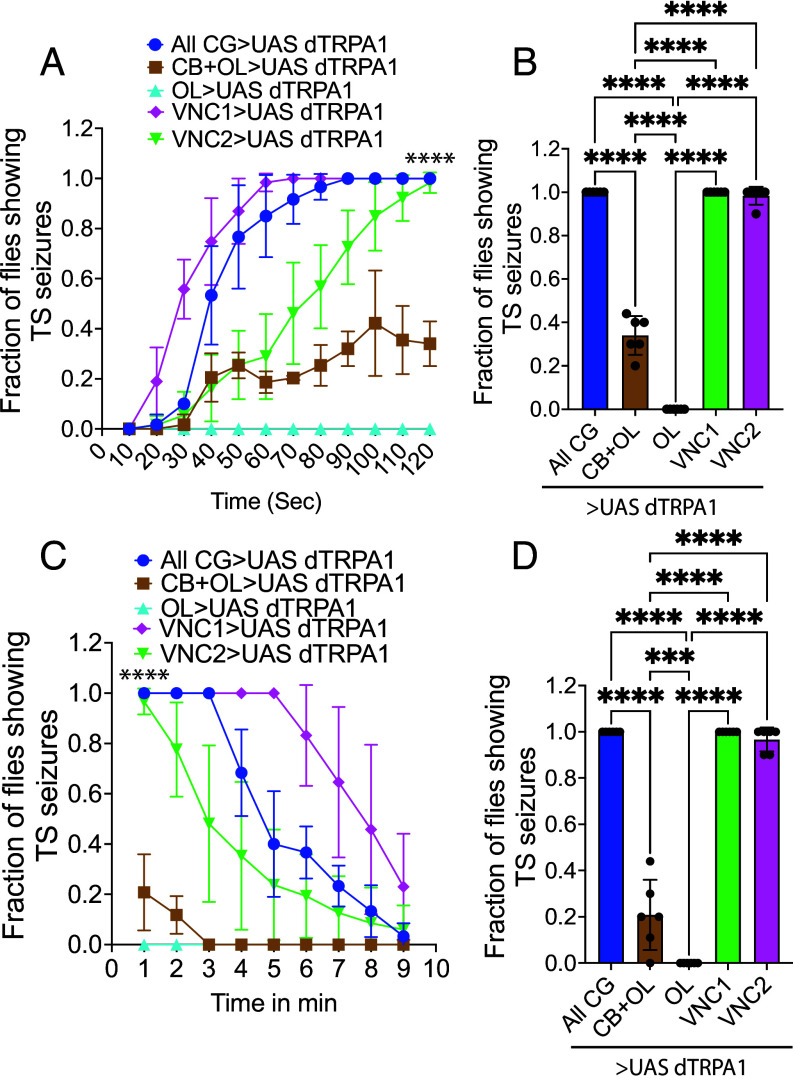
TRPA1 expression in the VNC-specific CG is sufficient to cause TS seizures in WT background. (*A*) Measurement of TS seizures in WT background when UAS TRPA1 is overexpressed in brain region–specific CG as a function of time. (*B*) Statistical analysis for the data in *A* at 120 s time point. (*C*) Quantification of heat-induced seizure recovery as a function of time in minutes. (*D*) Statistical analysis for the data in *C* at 1 min time point. Each data point in *A* and *C* represents a minimum of 50 flies from 6 independent experiments. Each dot in *B* and *D* represents an independent experiment. Error bars in *A*–*D* represent SD. The two way ANOVA multiple comparison was used to calculate *P* values where ****P ≤ 0.0001; ***P ≤ 0.001; **P ≤ 0.01; *P ≤ 0.05 and ns *P* > 0.05. All CG (R54H02-Gal4) CB+OL (R54H02-Gal4, R54D10-Gal80), OL (nsybGal80, R65B12Gal4).

Acute elevation of intracellular Ca^2+^ in CG, through overexpression of dTRPA1 triggers TS seizures in flies (31). The dTRPA1 is a heat activated cation channel that is normally expressed in a subset of thermosensitive neurons (46, 47). However, when expressed ectopically it promotes Ca^2+^ influx at temperatures above 26 °C but remains inactive at lower temperatures (46–48), suggesting dTRPA1 is a useful tool to acutely modulate intracellular calcium levels in vivo to induce heat sensitive seizures. Based on our findings in *zyd^1^* mutants, we questioned whether expressing UAS-dTRPA1 in the VNC-specific CG would be sufficient to induce TS seizures in a WT background. To test this hypothesis, we overexpressed UAS-dTRPA1 using two VNC-specific CG drivers and exposed 3- to 5-d-old adult flies to higher temperature. Interestingly, the expression of UAS-dTRPA1 in VNC+SEG+OL alone was sufficient to strongly induce TS seizures (Fig. 6 *A* and *B* and Movie S7). VNC1-Gal4 mediated expression of UAS-dTRPA1 in the VNC showed a stronger seizure phenotype compared to VNC2-Gal4, likely due to its stronger expression and or its expression in other types of glia such as ensheathing glia (Fig. 6 *A* and *B* and Movie S7). To further validate these findings, we expressed UAS-dTRPA1 in all CG using Wrapper-Gal4 and subsequently repressed its expression in VNC using R54D10-Gal80. As expected, expression of UAS-dTRPA1 in all CG strongly induced seizures at higher temperatures (Fig. 6 *A* and *B*). Repression of UAS-dTRPA1 expression in the VNC+SEG+OL significantly suppressed TS seizures (Fig. 6 *A* and *B* and Movie S7). Recovery analysis showed that flies expressing UAS-dTRPA1 in all CG, or VNC+SEG+OL required more time to recover compared to CB-specific expression (Fig. 6 *C* and *D*). Nevertheless, a small but a significant number of CB-specific UAS-dTRPA1 expressing flies show seizures; to investigate this phenotype further we analyzed single-fly behavior in more detail (*SI Appendix*, Fig. S11 *A* and *B*). About 38% of the flies showed complete seizures, 47% flies showed brief seizures lasting for only a few seconds, and 15% showed no seizures during the assay. Among flies that showed brief seizure, the time to seize greatly varied (20 to 120 s) with short seizure periods (10 to 40 s), and variable refractory periods (10 to 70 s) during the assay (*SI Appendix*, Data S2). Combined analysis of these individual fly behavioral data largely aligned with population analysis (Fig. 6 *A* and *C* and *SI Appendix*, Fig. S11 *A* and *B*). These data suggest that CB-specific CG expression of UAS-dTRPA1 is also able to induce seizures, however, that the seizures were transient in nature, and recover quickly (<1 min). In contrast, VNC-specific UAS-dTRPA1 expression caused complete seizures and took longer times to recover (3 to 13 min). These observations suggest that VNC-specific CG expression of UAS-dTRPA1 sensitizes flies to TS seizure more strongly than CB-specific expression. OL-specific expression (nsybGa80, R65B12Gal4) of UAS-dTRPA1 alone did not induce notable TS seizures (Fig. 6).

Taken together our results suggest that VNC-specific CG is a major contributor to prevent TS seizures.

### CB Function Is Dispensable for TS Seizures in *zyd^1^* Mutants.

Adult *Drosophila* sense warm temperatures via internal anterior cell (AC) neurons located inside the head and peripheral hot cell (HC) neurons located in the aristae. AC and HC neurons respond to innocuous temperatures (above 25 °C) via thermoreceptors TRPA1 and Gustatory Receptor [Gr28b (D)] respectively. Both these neurons project into distinct regions of the CB (49). Unlike adult flies, larvae respond to high temperatures via multiple dendritic (MD) neurons located in their body wall via two thermoreceptor TRP channels, including TRPA1 and Painless. TRPA1 and Painless respond to noxious warmth at temperatures above 39 °C. Although MD neurons are also found in adult abdomen (50), it is currently unknown if they have role in noxious warmth sensing. Nevertheless, our data from TS *zyd^1^* mutants, and the dTRPA1 overexpression analyses indicate that neuron glia interactions within the VNC+SEG region are critical for modulation of TS seizures. To investigate whether the CB function is necessary for TS seizures, we first ablated the antenna in *zyd^1^* mutants and performed TS assay. The ablation of antenna did not prevent seizure occurrence in *zyd^1^* mutants (*SI Appendix*, Fig. S11*C* and Movie S8). Further, we performed TS assay with decapitated *zyd^1^* mutants. Interestingly, headless *zyd^1^* flies continue to show seizure-like behavior (*SI Appendix*, Fig. S11*C* and Movie S9), suggesting TS seizures do not depend on CB function. Electrophysiological recordings also confirmed that decapitated *zyd^1^* mutant bodies show seizure-like activity upon heat stimulation (*SI Appendix*, Fig. S11*D*). Seizure-like behavior was also observed at high temperatures in decapitated flies overexpressing UAS-dTRPA1 in CG (*SI Appendix*, Fig. S11*C* and Movie S9).

It should be noted that *zyd^1^* mutants display seizures not only in response to temperature increase but also in response to any other elevated neuronal activity, such as vortexing to induce bang sensitivity or following recovering from a cold-induced state. It was suggested that any manipulation that increases the neuronal firing would lead to elevated extracellular K^+^ levels that are not properly buffered leading to seizures in *zyd^1^* mutants (29, 31). Indeed, we have observed that 2 wk old *zyd^1^* mutant flies displayed robust vortex-induced seizures, in addition to TS seizures (*SI Appendix*, Fig. S11*E*). However, previous studies showed that decapitation specifically suppressed vortex-induced seizures but not TS seizures in several other seizures mutants (51), indicating mechanical and temperature-induced seizures may involve anatomically distinct brain regions. Further, it was shown that classical bang sensitive mutants such as *eas^1^*, *jus,* and *bss^1^* do not show TS seizures (52). Consistently, we find that decapitation significantly suppressed vortex-induced seizures in *zyd^1^* mutant flies (*SI Appendix*, Fig. S11*E*), suggesting an important role of CB function. To further evaluate how vortex-induced seizures are regulated in *zyd^1^* mutants, we performed brain regional specific CG rescues. We found that CB-specific expression of UAS Zyd was sufficient to suppress vortex-induced seizures in *zyd^1^* mutants (*SI Appendix*, Fig. S11*F*). However, even VNC-specific CG expression of UAS Zyd, was also able to suppress vortex-induced seizures in *zyd^1^* mutants. It should be noted that VNC-specific CG rescues also partly expressed in OL and SEG of the CB (Fig. 1*I*). Together, these observations suggest that CB function may play an important role in vortex inducible seizures but is dispensable for TS seizures.

## Discussion

While glial heterogeneity across mammalian brain regions is well established (18–20), a lack of intersectional genetic strategies has limited our understanding of its functional significance (18, 19). Consequently, only a few studies demonstrated these contributes; for instance, in the mice spinal cord ventral horn specifically express axon guidance protein semaphorin3a (Sema3a), conditional deletion of which leads to selective loss of local α-motor neurons, and reduced peak strength (18, 53). In hypothalamic arcuate nucleus astrocytes modulate synaptic transmission in pro-opiomelanocortin (POMC) and agouti-related peptide (AgRP) expressing neurons. Deletion of astrocytic leptin receptor reduces membrane coverage, leading to leptin resistance and increased fasting or ghrelin-induced hyperphagia (54, 55).

Recent *Drosophila* single-cell transcriptional atlases revealed that glia display greater morphological than transcriptional diversity, though detection may obscure low-level transcripts (23). The existence of region-specific Gal4 lines support possible transcriptional heterogeneity (22). Using standard genetic tools, in this study, we optimize gene expression in CG subtypes across brain subregions and demonstrate that CG in different brain regions differentially regulate specific seizure types. We show that OL- and CB-specific CG are important in suppression of LI seizures, whereas VNC-specific CG are important in suppression of TS seizures. Although underlying mechanisms are currently unclear, we propose that CG provide local regulation to prevent occurrence of seizures.

*Drosophila* OL and CB constitute neuronal circuits that process visual information (56, 57) and *norpA*-mediated signaling is essential for LI seizures in *cpes* mutants (27). These mutants show severe defects in CG morphology and elevated neuronal activity during seizures (27). This study found that OL- and CB-specific CG are sufficient to suppress LI seizures highlighting critical local neuron–glia interactions. The absence of CG support likely impairs neuroblast proliferation (58–62), circuit wiring (59, 63), phagocytic clearance of dead neurons (64–68), and ion balance (28, 29).

Glial calcium signaling and calmodulin-dependent pathways regulate TS seizures (29, 31, 69). Acute elevation of CG calcium levels has been shown to trigger excessive neuronal activity leading to seizure-like activity (31). In *zyd^1^* mutants, elevated basal Ca^2+^ levels enhanced calcineurin-dependent endocytosis of K2P leak channel Sandman impairs K^+^ buffering, causing TS seizures (29, 31). In this study, we found that ZYD expression in VNC-specific CG is essential for suppression of TS seizures. Furthermore, acute influx of Ca^2+^ into VNC-specific CG via UAS-dTRPA1 expression was sufficient to cause TS seizures in WT flies. Headless *zyd^1^* mutant and UAS-dTRPA1 overexpressing flies confirms that VNC-specific CG is sufficient for regulation of TS seizures. Voltage-gated ion channels such as Paralytic (Para), and Shaker cognate I (Shal) are enriched in the VNC, and axon initial segments of motor neurons, suggesting glia–neuron interactions support rapid action potential conduction in these regions (70, 71). Headless flies expressing dTRPA1 in astrocyte-like glia become paralyzed at 33 °C, suggesting acute Ca^2+^ influx-induced paralysis does not require CB function (69). Because the *Drosophila* VNC can drive many motor behaviors in headless flies (72), it is also likely to sense higher temperatures, and depends on normal glial function to regulate neuronal activity. However, it should be noted that higher temperatures broadly affect ion channel kinetics, a CB role in TS seizures cannot be fully excluded, as electrophysiological and behavioral analysis cannot assess focal seizures in flies. Nevertheless, the fact that *zyd^1^* mutants or UAS dTRPA1 overexpression in VNC are significantly lowering the temperature threshold needed for induction of seizures, and that the rescues increase the seizure threshold back to WT levels suggests that defects in thoracic glia is sufficient for TS seizures. Because most motor circuits driving the behavior including DLM motoneurons, reside in the thoracic ganglion, glia in this region may directly regulate the TS seizure threshold. In contrast, CB may contribute more strongly to vortex-induced seizures in *zyd^1^* mutants, as decapitation significantly reduced these seizures. This is consistent with previous findings that decapitation suppresses vortex-induced seizures in bang-sensitive mutants but not TS seizures in temperature-sensitive mutants, suggesting distinct seizure foci in these mutant classes (51, 52). Future studies should compare these behaviors across various seizures mutants to clarify the underlying neuronal mechanisms.

Together, our brain region–specific rescue approach provides valuable insights into the local role of CG in seizures and supports further study of localized neuron–glia interactions in broader behavioral outcomes.

## Materials and Methods

A detailed description of materials and methods is provided in *SI Appendix*.

### Fly Stock and Husbandry.

c*pes* mutants and UAS-Cpes were generated in the lab and reported before (27). Nrv2-p65(AD), and Wrapper-Gal4.DBD are kind gifts from (Coutinho-Budd) (21). *zyd^1^* mutants, UAS-Zyd are kind gifts from (Troy Littleton) (31). Standard cloning and transgenesis practices were employed in generating some of the stocks described in this study including R54D10-Gal4.DBD, R54D10-nlsLexA.p65, R54D10-Gal80, R9F07-Gal4.DBD, R9F07-Gal80, UAS-Cpes-HA, and UAS-Zyd-HA and described in *SI Appendix*. All other stocks were from Bloomington Drosophila stock center and described in *SI Appendix*.

### Immunohistochemistry and Confocal Microscopy.

Immunostaining was performed as described before (27, 73). They are described in *SI Appendix*.

### Light- and Temperature-inducible Seizures.

The assay for LI seizures and TS seizures were performed as described previously and detailed in *SI Appendix* (27, 29, 31).

### Seizure Recordings from *Drosophila* Flight Muscles.

Electrophysiological recordings from the Dorso-Longitudinal Muscle of the GF circuit have been described in detail previously (39–41, 74) and detailed in *SI Appendix*.

### Negative Geotaxis Assay and Single Fly Walking Assay.

Negative geotaxis assay was performed as described previously (75) and the single fly walking assay is detailed in *SI Appendix*.

## Supplementary Material

Appendix 01 (PDF)

Movie S1. **Light induces partial or complete seizures in *cpes* mutants.** Individual *cpes* mutant flies showing partial or complete seizures in response to light are shown. All individual movies were assembled and edited into single sequence using Adobe Premiere pro software. Video shown here is the 3x speed of real time.

Movie S2. **Central brain specific cortex glia plays important role in suppression of light inducible seizures in *cpes* mutants.** Expression of UAS-Cpes in VNC specific cortex glia do not suppress light inducible seizures. Whereas expression of UAS Cpes in CB and OLs significantly suppressed light inducible seizures. **CB+VNC1**=Nrv2-p65(AD), VT038983-Gal4.DBD>UAS Cpes; **OL1**=nSybGal80, R9F07Gal80, R65B12-Gal4>UAS Cpes; **VNC1**=Nrv2-p65(AD), R54D10-Gal4 DBD> UAS Cpes; **VNC2**=VT038983-p65(AD), R54D10-Gal4.DBD>UAS Cpes; **CB1**=Nrv2-p65(AD), VT038983-Gal4.DBD> UAS Cpes, R54D10-nlsLexAp65>13xLexAOP2 Kzip+; ***cpes***=UAS Cpes alone. All the above-mentioned rescues are in *cpes* mutant background. All individual movies were assembled and edited into single sequence using Adobe Premiere pro software. Video shown here is the 3x speed of real time.

Movie S3. **The *cpes* mutants do not show robust temperature induced seizures.** Individual *cpes* mutant flies previously confirmed to show complete light inducible seizures were subsequently subjected to temperature sensitive assay by submerging fly vials in a water bath maintaining at temperature 38.5 °C. All individual movies were assembled and edited into single sequence using Adobe Premiere pro software. Video shown here is the 3x speed of real time.

Movie S4. **Ventral nerve cord specific cortex glia plays important role in suppression of temperature sensitive seizures in *zyd*^1^ mutants.** Expression of UAS-Zyd in VNC specific cortex glia is sufficient to suppress temperature sensitive seizures in *zyd*^1^ mutants. **CB+VNC1**=Nrv2-p65(AD), VT038983-Gal4.DBD>UAS-Zyd; **OL**= R65B12-Gal4> UAS-Zyd; **VNC2**=VT038983-p65(AD), R54D10-Gal4.DBD> UAS-Zyd; **CB1**=Nrv2-p65(AD), VT038983-Gal4.DBD> UASZyd, R54D10-nlsLexAp65>13xLexAOP2 Kzip+; *zyd*^1^= UAS-Zyd alone. All the above-mentioned rescues are in *zyd*^1^ mutant background. All individual movies were assembled and edited into single sequence using Adobe Premiere pro software. Video shown here is the 3x speed of real time.

Movie S5. **ZYD is required in VNC for the suppression of temperature sensitive seizures in *zyd*^1^ mutants.** Expression of ZYD in all cortex glia (All CG) fully suppress temperature sensitive seizures. However, temperature sensitive seizures reoccurred upon repression of Zyd expression in VNC when co-expressed with R54D10-Gal80 in All CG rescue background. *zyd*^1^=UAS-Zyd alone; All CG=R54H02-Gal4>UAS-Zyd; CB+OL=R54H02-Gal4, R54D10-Gal80>UAS-Zyd. All the above-mentioned rescues are in *zyd*^1^ mutant background. All individual movies were assembled and edited into single sequence using Adobe Premiere pro software. Video shown here is the 3x speed of real time.

Movie S6. **Single fly walking assay at high temperature.** Individual flies in a 60x15mm petri dish were submerged into a water bath maintaining at 38.5 °C, video was captured and walking was tracked as described in the methods section. *zyd*^1^= UAS-Zyd; All CG=R54H02-Gal4>UAS-Zyd; CB1=Nrv2-p65(AD), VT038983-Gal4.DBD> UAS-Zyd, R54D10-nlsLexAp65>13xLexAOP2 Kzip+; VNC2=VT038983-p65(AD), R54D10-Gal4.DBD> UAS-Zyd CB+OL=R54H02-Gal4, R54D10-Gal80>UAS-Zyd. All individual movies were assembled and edited into single sequence using Adobe Premiere pro software. Video shown here is the 3x speed of real time.

Movie S7. **Acute Ca2+ influx into VNC specific cortex glia is sufficient to induce temperature sensitive seizures in wild type flies.** UAS-dTRPA1 is overexpressed in cortex glia in different parts of the brain including VNC, CB and OL. dTRPA1 gets activated only at temperatures above 25°C. Expression and activation dTRPA1 in VNC specific cortex glia is sufficient to induce temperature sensitive seizures in wild type flies. All CG=R54H02-Gal4>UAS-dTRPA1; CB+OL=R54H02-Gal4, R54D10-Gal80>UAS-dTRPA1; OL=nsybGal80, R65B12 Gal4>UAS-dTRPA1; VNC1=Nrv2-p65(AD), R54D10-Gal4 DBD> UAS-dTRPA1; VNC2=VT038983- p65(AD), R54D10-Gal4.DBD>UAS-dTRPA1. All individual movies were assembled and edited into single sequence using Adobe Premiere pro software. Video shown here is the 3x speed of real time.

Movie S8. **Aristae are dispensable for initiation of temperature sensitive seizures.** Aristae ablated *zyd*^1^ mutant flies were subjected to heat shock in a water bath maintaining temperature at 38.5°C for 2 min. Video shown here is the 3x speed of real time.

Movie S9. **Central brain function is dispensable for initiation of temperature sensitive seizures.** *zyd*^1^ mutant flies were decapitated under CO2 and allowed recovered for 1 hour. Flies were transferred to a Petri dish (60x15mm) and sealed with parafilm. Subsequently, decapitated flies in the dish were immersed in a water bath maintaining temperature at 38.5°C for 2 min to measure temperature sensitivity. VNC=VT038983-p65(AD), R54D10-Gal4.DBD>UAS-dTRPA1. Video shown here is the 3x speed of real time.

## Data Availability

All study data are included in the article and/or supporting information and the fly stocks generated in the study are available upon request from corresponding authors. There is no new data associated with this article.
