# Supplementary material for: Light- and temperature-sensitive seizures are regulated by spatially distinct cortex glial populations in the central nervous system
Source: Proc Natl Acad Sci U S A. 2026 Jul 21;123(30):e2605750123. doi: 10.1073/pnas.2605750123 (PMC13417042; doi:10.1073/pnas.2605750123)
Supplement: Supplementary file 1 — Appendix 01 (PDF) [file pnas.2605750123.sapp.pdf]

PNAS

Supporting Information for

Light- and temperature-sensitive seizures are regulated by spatially distinct cortex glial populations in the central nervous system

Govind Kunduri[1*], Tanja Angela Godenschwege[2], Katherine Sankey[2], Kandahalli Venkataranganayaka Abhilasha[1], Usha Acharya[1*] and Jairaj K. Acharya[1*]

[1]Cancer and Developmental Biology Laboratory, National Cancer Institute, Frederick, MD 21702.

[2]Biological Sciences Department, Florida Atlantic University, Jupiter, FL 33458.

[*]Corresponding Authors

Kunduri Govind

kunduri.govind@nih.gov

Usha Acharya

Usha.acharya@nih.gov

Jairaj K Acharya

acharyaj@mail.nih.gov

Contact:

Jairaj K Acharya

Rm. 22-90B, Bldg. 560

National Cancer Institute

1050 Boyle Street

Frederick, MD 21702

Ph: 301-846-7051

This PDF file include

Detailed materials and methods

Captions for Movies S1 to S9

Fig.S1 to S11

Tables S1 to S3

Dataset S1 to S2

SI References

Other supplementary materials for this manuscript include the following:

Movies S1 to S9

Materials and methods

Fly stocks and husbandry

The following stocks were obtained from Bloomington *Drosophila* stock center (BDSC), including, VT038983-p65(AD) (BDSC#72320), VT038983-Gal4.DBD (BDSC#72724), R65B12-Gal4 (BDSC#39342), nSyb-Gal80 (BDSC#92154), R54H02-Gal4 (BDSC#45784), UAS-TRPA1 (BDSC#26264), LexAop-rCD2-GFP (BDSC#66544), 13x LexAop-KZip+.3xHA (BDSC#76253), UAS-mCD8GFP (BDSC#5130), UAS-mCherry-NLS (BDSC#38424), Wrapper-nlsLexA::p65 (BDSC#94730),

c*pes* mutants and UAS Cpes were generated in the lab and reported before (1).

Nrv2-p65(AD), and Wrapper-Gal4.DBD are kind gifts from (Coutinho-Budd) (2).

zyd^{1} mutants, UAS-Zyd are kind gifts from (Troy Littleton) (3).

Stocks generated in this study include R54D10-Gal4.DBD, R54D10-nlsLexA.p65, R54D10-Gal80, R9F07-Gal4.DBD, R9F07-Gal80, UAS-Cpes-HA, and UAS-Zyd-HA.

Cloning and transgenesis

R54D10 and R9F07 enhancers were PCR amplified using primers described at Janelia Flylight project website. These primers were designed to be compatible with gateway cloning technology (Primers Table 3). The PCR product was first cloned into pDONR 221 using BP clonase (Cat#11789020), subsequently cloned into destination vectors pBPZPGal4DBD (Addgene#26233), pBPGal80UW6 (Addgene#26236), and pBPnlsLexA p65UW, (Addgene #26230) using LR clonase reaction as described by manufacturer (Thermofisher Scientific Cat#11791020). For cloning UAS Cpes-HA and UAS Zyd-HA, we have first PCR amplified respective cDNAs with primers described in Table 3. The PCR products were first cloned into pDONR 221 using BP clonase and subsequently cloned into pTWH vector using LR clonase reaction. Sequence confirmed clones were sent out for embryo injection service at BestGene Inc.

Immunohistochemistry

Drosophila CNS from larvae, pupae and adults were dissected as described before (https://www.janelia.org/project-team/flylight/protocols and (4). Immunostaining of the dissected CNS was carried out as described previously (1). Briefly, fly brains were dissected in a glass well plate with phosphate buffered saline (1x) and fixed with 4% paraformaldehyde (PFA, EMS Cat#15713-S) in PBS for 1 hour at room temperature (RT). Fixed brains were permeabilized by washing with 1xPBS containing 0.1% Triton x100 (PBST) for 3 times each with 20 min interval at RT (25°C) and blocked with 5% normal goat serum (NGS, Gibco REF#16210-064) in PBST for 1 hour at RT. Subsequently, brains were incubated with primary antibody (10µg/ml in 5% NGS, PBST) for 12 hours/overnight at 4°C and followed by brains were washed with PBST (3 times each with 20 min interval at RT). Brains were then incubated with secondary antibody (10µg/ml in 5% NGS PBST) for 12hours/overnight at 4°C and washed with PBST 3 times each with 20 min interval at RT. Brains were stained with DAPI (1:2000 dilution of 10mg/ml stock, Cat#62247) for 10 min at RT, followed by final wash with PBST for 10 min at RT. In the last step all the buffer was removed by pipetting, and a drop of mounting media (Vectashield REF#H1000) is added. The brains were then mounted on a glass slide (VWR Cat#48311-703) with SecureSeal imaging spacer (GBL654008) and covered with cover slip (VWR Cat#48393081/Cat#16004-094) and sealed with clear nail polish (Sally Hansen). The anti-GFP rabbit polyclonal antibody and mCherry monoclonal antibody were from Novus biologicals (NB600-300) and Takara (Cat#632543) respectively. HA-Tag (C29F4) Rabbit mAb, and HA-Tag (6E2) mouse mAb are from Cell Signaling.

Confocal microscopy

For Fig.1, Fig.2, Fig. 5, Fig.S1, Fig.S2 and Fig.S3, immunostained slides were imaged using ZEISS confocal laser scanning microscope LSM880. ZEN black software was used for visualization and acquisition of images, wherein laser settings were optimized and kept constant across all the samples. Each sample was imaged with the Z-scan thickness 40-50 µm (2 µm intervals), and the tile scan was set up to 3x3 (3rd instar brains) or 4x4 (pupa and adult brains) with 40x oil immersion objective. Tiles of each image were automatically stitched by ZEN black software. Subsequent stitched images were analyzed by FIJI (ImageJ) software, where images were represented as Z-projections.

For Fig.S4, and Fig.S8 immunostained slides were imaged using Andor Spinning disk confocal microscopy. Fusion software was used to visualize and acquire images. Each sample was imaged with 40x oil immersion objective, Z-scan thickness of 80 μm (1μm intervals) and a custom tile scan to cover sample area and imaged at constant laser settings across samples. Images were stitched automatically on fusion software, and the final images were further analyzed using Imaris software or FIJI and shown as 3D projections.

Light inducible seizures

The assay for light inducible seizures was performed as described previously (1). Briefly, *cpes* mutants were raised at room temperature (20-22°C) and aged at 25°C with 12 hours light and 12 hours dark cycles for 3-4 weeks. Each food vial contained 20-30 flies (population assay), and they were then transferred to new vial every two days until the desired age. For single fly analysis, each food vial contained one fly. The day before the assay flies were allowed to adapt to the dark in a black box (12 hours/overnight). Next day, under infra-red light individual fly vials were arranged on a white cardboard box and videos were captured in real time using Sony cyber shot DSCHX400V camera, 3 seconds under infrared light and 2-5 min under white light (GE, LED, Daylight, 100w/15w, 1600 lumens). Up to 100 flies were tested for each genotype and percent or fraction of flies showing light inducible seizures/photosensitive epilepsy was plotted. We have quantified two different behavioral phenotypes that *cpes* mutant flies exhibited upon exposure to light. Those include, flies fell down lying on their back or side (complete seizure), and flies fell down but remain standing on their legs without any movement for at least 20 seconds (partial seizure). Both behaviors were scored manually by assessing the videos at every 20 seconds interval and fraction of flies in seizure was scored. An average fraction of flies showing seizures from a minimum of 3 independent experiments were plotted at each time interval.

Temperature sensitive seizures

The assay for temperature sensitive seizures was performed as described previously (3, 5). Briefly, adult male flies were transferred in groups of 10-20 per vial (population assay) or single fly in each vial (single fly assay) and aged for 3-5 days. On the day of the assay, flies were transferred to empty vial and allowed to rest for 1-2 hours, subsequently each vial was immersed into preheated transparent water bath maintaining temperature at 38.5°C for 2 minutes for monitoring seizure

induction and 2-10 min outside the water bath at RT (22°C) for monitoring seizure recovery. Videos were captured using real time using Sony cyber shot DSCHX400V camera. Total number of flies tested in all assays was always more than 40. To analyze temperature induced seizure behavior, we have quantified only flies that show complete seizures, that is, flies are constantly lying on their back or side with legs twitching. Flies walking up and down on the walls were considered wild type or rescue behavior. Seizure measurements were taken at every 10 second interval and plotted as a fraction of flies showing heat induced seizures as a function of time.

Seizure recordings from Drosophila flight muscles.

Electrophysiological recordings from the Dorso-Longitudinal Muscle (DLM) of the giant fiber (GF) circuit have been described in detail previously (6). In brief, tungsten stimulating and ground electrodes were placed in the head and abdomen, respectively. Glass recording electrodes were inserted into the left and right DLMs for recording of responses in the flight motoneurons. However, modifications to mounting and the recording protocols were made to adjust for the light and temperature induced seizure assays.

For light-induced seizures, animals were 24 hrs dark-adapted in food vials. Subsequently, the animals were briefly anesthetized with CO_2 and mounted in dental wax under red-light (HinsGEAR LED Red flashlights). After a 60 min minimum rest period in the dark in light proof containers with wet kimwipes for humidification, electrodes were placed into the animals under red-light (HinsGEAR LED Red flashlights). Single pulse stimulations of the GF circuit were used to ensure proper recordings from the DLMs. After another 10-15 mins rest period in the dark, passive (no stimulation of GF circuit in the brain) or active (with 0.5 Hz brain stimulations) recordings were obtained in the dark for varying length (1-15 min) depending on the presence or absence of seizures in the dark. Active recordings allow to ensure proper recording electrode placement in the DLMs throughout the assay, as well as to assess for potential synaptic failures in the GF to DLMn circuit after seizures (7-9). Finally, a baseline recording was obtained in the dark for 30-60 secs before light onset for 60secs with 2000-5000k lumen flashlights (Wurkkos WK20S or Infinity X1). Some animals were retested after a minimum of a 1 min rest period in the dark. The traces of the electrophysiological recordings were assessed in Clampex 11.42 software, with seizures being defined as a minimum burst of 10 muscle action potentials with a minimum firing rate of 10Hz.

For temperature-induced seizures, animals were briefly anesthetized with CO_2, mounted in dental wax, and rested for a minimum of 60 min in ambient light conditions in a large petri dish with wet kimwipes for humidification. After electrode placements, single pulse stimulation of the GF circuit was used to ensure proper recordings from the DLMs. Subsequently a baseline recording with 0.5 Hz brain stimulations was obtained at room temperature (21-23°C) for 30-60 secs. From a distance of approximately 70 -100 cm a 1800W SEEKONE Heat Gun was used to raise the temperature around the animals. A UEi PDT655 Differential Temperature Folding Pocket Thermometer placed next to animals was used to monitor the temperature. The temperature was raised to 38.5°C and kept at that temperature (+/- 2.5°C) for a minimum of 60 secs, unless a seizure started at a lower temperature, in which case we recorded seizures onset temperature and continued heating for an additional 10-30 secs.

Single fly walking analysis

Single flies of appropriate age from each genotype were anesthetized under CO_2 and transferred to 60x15mm polystyrene petri dish and allowed to recover and acclimatize for 1 hour at room temperature. Subsequently, petri dish with single fly was immersed in water bath maintained at 38.5 °C and video was recorded for 2 min and 30 seconds using Canon HD CMOS (VIXIA HF R800 HD) camera. Videos were cropped using imovie software and converted to uncompressed raw .avi files using ffmpeg software using the following command ffmpeg -i input.mp4 -mp4 -c:v rawvideo output.avi. The .avi files were opened using FIJI (imagej) software and analyzed using manual tracking plugin, wherein movement of fly in each video frame was manually traced by clicking on the fly. In the settings of the plugin, time interval for each frame is calculated by 1/30 (30 frames per second) and the XY calibration is calculated from set scale option by entering known diameter of the dish, which will give value in pixels/cm. During tracking, fly movement in each frame was displayed in µm per frame. The distance moved in 30 frames taken as movement in 1 second (calculated in excel using functions =wraprows and SUM) and the data is plotted as distance walked per second or average distance walked in first or last 60 seconds of the assay using Prism 10 software.

Negative geotaxis assay

Negative geotaxis assay was performed as described previously (10), briefly, flies of appropriate genotype were sorted in groups of 10 under CO_2 anesthesia transferred to fresh fly food vial and

allowed to age for 5-7 days. One the day of assay climbing apparatus was prepared for each group by joining two empty vials facing each other by tape. The openings of the vials were aligned perfectly to avoid uneven climbing. Vertical distance of 8cm was measured and marked on the lower vial. Groups of 10 flies were transferred to lower vial and immediately covered with top vial and tape. Flies were allowed to acclimatize to the vials for 30 min and assay was performed by gently taping down to the bottom of the vial and measure the number of flies cross the 8cm mark by 10 sec.

Captions for Movies

Movie.1. Light induces partial or complete seizures in *cpes* mutants. Individual *cpes* mutant flies showing partial or complete seizures in response to light are shown. All individual movies were assembled and edited into single sequence using Adobe Premiere pro software. Video shown here is the 3x speed of real time.

Movie.2. Central brain specific cortex glia plays important role in suppression of light inducible seizures in *cpes* mutants. Expression of UAS-Cpes in VNC specific cortex glia do not suppress light inducible seizures. Whereas expression of UAS Cpes in CB and OLs significantly suppressed light inducible seizures. **CB+VNC1**=Nrv2-p65(AD), VT038983-Gal4.DBD>UAS Cpes; **OL1**=nSybGal80, R9F07Gal80, R65B12-Gal4>UAS Cpes; **VNC1**=Nrv2-p65(AD), R54D10-Gal4 DBD> UAS Cpes; **VNC2**=VT038983-p65(AD), R54D10-Gal4.DBD>UAS Cpes; **CB1**=Nrv2-p65(AD), VT038983-Gal4.DBD> UAS Cpes, R54D10-nlsLexAp65>13xLexAOP2 Kzip+; ***cpes***=UAS Cpes alone. All the above-mentioned rescues are in *cpes* mutant background. All individual movies were assembled and edited into single sequence using Adobe Premiere pro software. Video shown here is the 3x speed of real time.

Movie.3. The *cpes* mutants do not show robust temperature induced seizures. Individual *cpes* mutant flies previously confirmed to show complete light inducible seizures were subsequently subjected to temperature sensitive assay by submerging fly vials in a water bath maintaining at temperature 38.5 °C. All individual movies were assembled and edited into single sequence using Adobe Premiere pro software. Video shown here is the 3x speed of real time.

Movie.4. Ventral nerve cord specific cortex glia plays important role in suppression of temperature sensitive seizures in *zyd*^{1} mutants. Expression of UAS-Zyd in VNC specific cortex glia is sufficient to suppress temperature sensitive seizures in *zyd*^{1} mutants. **CB+VNC1**=Nrv2-p65(AD), VT038983-Gal4.DBD>UAS-Zyd; **OL**= R65B12-Gal4> UAS-Zyd; **VNC2**=VT038983-p65(AD), R54D10-Gal4.DBD> UAS-Zyd; **CB1**=Nrv2-p65(AD), VT038983-Gal4.DBD> UAS-Zyd, R54D10-nlsLexAp65>13xLexAOP2 Kzip+; *zyd*^{1}= UAS-Zyd alone. All the above-mentioned

rescues are in zyd^1 mutant background. All individual movies were assembled and edited into single sequence using Adobe Premiere pro software. Video shown here is the 3x speed of real time.

Movie.5. ZYD is required in VNC for the suppression of temperature sensitive seizures in zyd^1 mutants. Expression of ZYD in all cortex glia (All CG) fully suppress temperature sensitive seizures. However, temperature sensitive seizures reoccurred upon repression of Zyd expression in VNC when co-expressed with R54D10-Gal80 in All CG rescue background. zyd^1=UAS-Zyd alone; All CG=R54H02-Gal4>UAS-Zyd; CB+OL=R54H02-Gal4, R54D10-Gal80>UAS-Zyd. All the above-mentioned rescues are in zyd^1 mutant background. All individual movies were assembled and edited into single sequence using Adobe Premiere pro software. Video shown here is the 3x speed of real time.

Movie.6. Single fly walking assay at high temperature. Individual flies in a 60x15mm petri dish were submerged into a water bath maintaining at 38.5 °C, video was captured and walking was tracked as described in the methods section. zyd^1= UAS-Zyd; All CG=R54H02-Gal4>UAS-Zyd; CB1=Nrv2-p65(AD), VT038983-Gal4.DBD> UAS-Zyd, R54D10-nlsLexAp65>13xLexAOP2 Kzip+; VNC2=VT038983-p65(AD), R54D10-Gal4.DBD> UAS-Zyd CB+OL=R54H02-Gal4, R54D10-Gal80>UAS-Zyd. All individual movies were assembled and edited into single sequence using Adobe Premiere pro software. Video shown here is the 3x speed of real time.

Movie.7. Acute Ca2+ influx into VNC specific cortex glia is sufficient to induce temperature sensitive seizures in wild type flies. UAS-dTRPA1 is overexpressed in cortex glia in different parts of the brain including VNC, CB and OL. dTRPA1 gets activated only at temperatures above 25°C. Expression and activation dTRPA1 in VNC specific cortex glia is sufficient to induce temperature sensitive seizures in wild type flies. All CG=R54H02-Gal4>UAS-dTRPA1; CB+OL=R54H02-Gal4, R54D10-Gal80>UAS-dTRPA1; OL=nsybGal80, R65B12 Gal4>UAS-dTRPA1; VNC1=Nrv2-p65(AD), R54D10-Gal4 DBD> UAS-dTRPA1; VNC2=VT038983-p65(AD), R54D10-Gal4.DBD>UAS-dTRPA1. All individual movies were assembled and edited into single sequence using Adobe Premiere pro software. Video shown here is the 3x speed of real time.

Movie.8. Aristae are dispensable for initiation of temperature sensitive seizures. Aristae ablated *zyd*^{1} mutant flies were subjected to heat shock in a water bath maintaining temperature at 38.5°C for 2 min. Video shown here is the 3x speed of real time.

Movie.9. Central brain function is dispensable for initiation of temperature sensitive seizures. *zyd*^{1} mutant flies were decapitated under CO_2 and allowed recovered for 1 hour. Flies were transferred to a Petri dish (60x15mm) and sealed with parafilm. Subsequently, decapitated flies in the dish were immersed in a water bath maintaining temperature at 38.5°C for 2 min to measure temperature sensitivity. VNC=VT038983-p65(AD), R54D10-Gal4.DBD>UAS-dTRPA1. Video shown here is the 3x speed of real time.

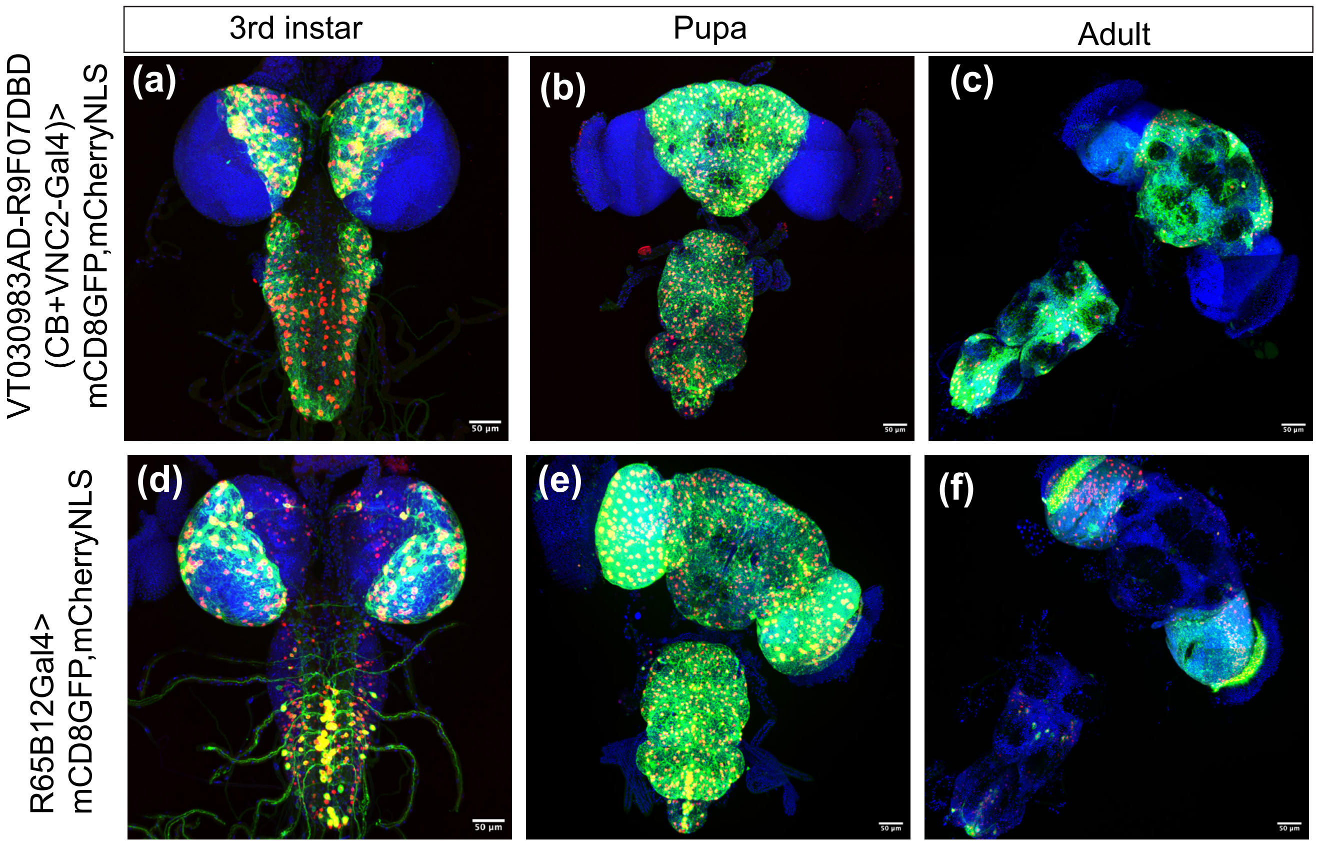

3rd instar
Pupa
Adult
VT030983AD-R9F07DBD
(CB+VNC2-Gal4)>
mCD8GFP,mCherryNLS
R65B12Gal4>
mCD8GFP,mCherryNLS
(a)
(b)
(c)
(d)
(e)
(f)
50 µm

Fig.S1. (a-c) Expression pattern of additional CB and VNC specific cortex glial split-Gal4 driver VT030983-p65(AD), R9F07-Gal4.DBD>mCD8GFP, mCherryNLS in 3rd instar CNS (a), 48 hours post pupation CNS and 7-day old adult fly CNS. **(d-f)** Expression pattern of R65B12-Gal>mCD8GFP, mCherryNLS in 3rd instar CNS, 48 hours post pupation CNS, and 7-day old adult CNS. Scale bar in each of the image corresponds to 50μm.

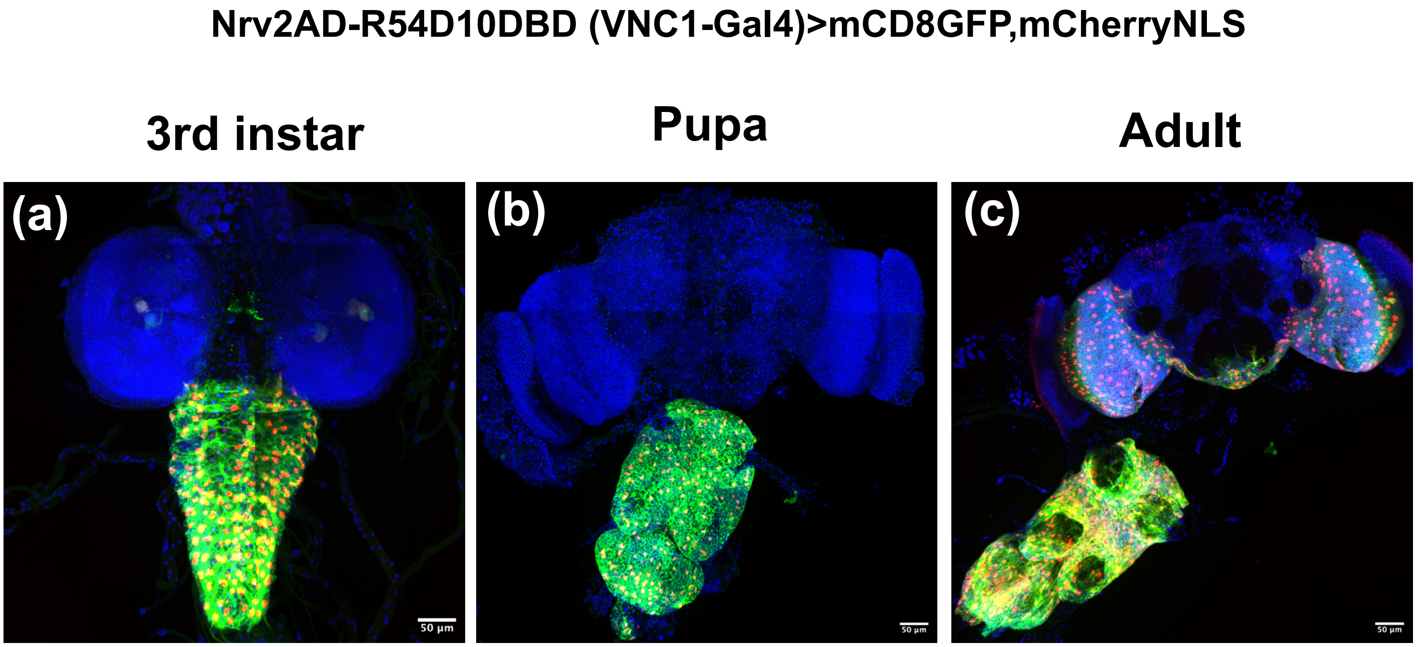
Nrv2AD-R54D10DBD (VNC1-Gal4)>mCD8GFP,mCherryNLS
3rd instar
Pupa
Adult
(a)
(b)
(c)
50 μm
50 μm
50 μm

Fig.S2. Expression patterns of additional VNC cortex glial specific split Gal4 drivers. **(a-c)** expression pattern of splitGal4 driver Nrv2-p65(AD), R54D10-Gal4.DBD>mCD8GFP, mCherryNLS in 3rd instar CNS (a), 48 hours post pupation CNS (b), and 7-day old adult CNS (c). Scale bar in each of the image corresponds to 50μm.

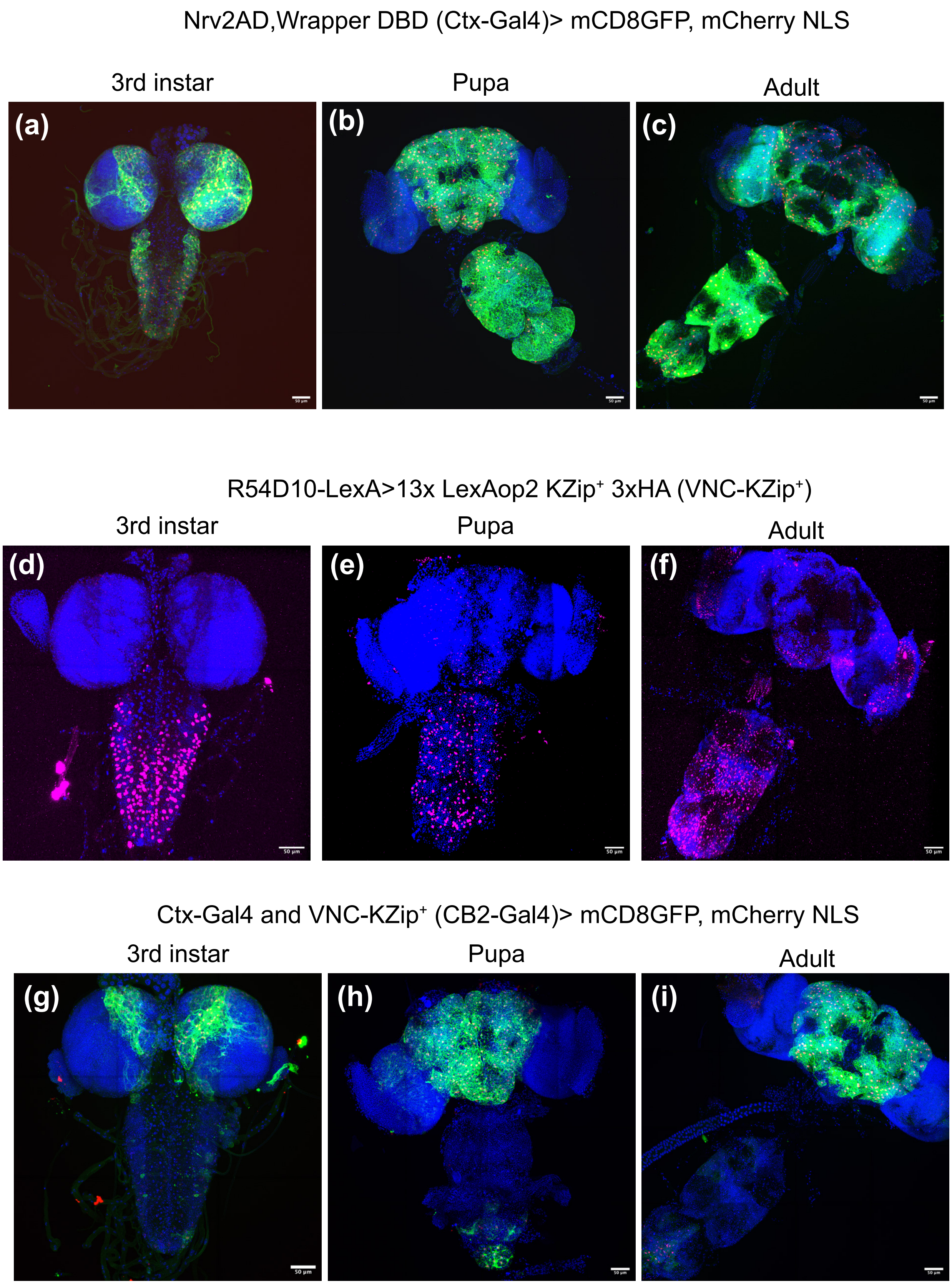
Nrv2AD,Wrapper DBD (Ctx-Gal4)> mCD8GFP, mCherry NLS
3rd instar
Pupa
Adult
(a)
(b)
(c)
R54D10-LexA>13x LexAop2 KZip⁺ 3xHA (VNC-KZip⁺)
3rd instar
Pupa
Adult
(d)
(e)
(f)
Ctx-Gal4 and VNC-KZip⁺ (CB2-Gal4)> mCD8GFP, mCherry NLS
3rd instar
Pupa
Adult
(g)
(h)
(i)

Fig.S3. (a-c) Expression pattern of previously described split Gal4 that generically express in all cortex glia, Nrv2-p65(AD), Wrapper-Gal4.DBD (Ctx-Gal4) in 3rd instar CNS (a), 48 hours post pupation CNS (b), and 7-day old adult CNS (c). Ctx-Gal4 drives the expression of mCD8GFP and mCherry NLS. **(d-f)** expression pattern of R54D10-nlsLexAp65 driving the expression of 13x LexAop2-KZip+ 3xHA. Immunostaining was performed with rabbit anti HA antibody and Alexa Fluor 647 conjugated secondary antibody. 3rd instar CNS (d), 48 hours post pupation CNS (e) and 7-day old adult CNS (f). **(g-i)** CB specific expression pattern was optimized by crossing Ctx-Gal4 with killer zipper system in the VNC using R54D10-nlsLexAp65 and 13xLexAop2-KZip+. The reporters used here are mCD8GFP and mcherry NLS in 3rd instar CNS (g), 48 hours post pupation CNS (h) and 7-day old adult CNS (i). Scale bar in each of the image corresponds to 50μm.

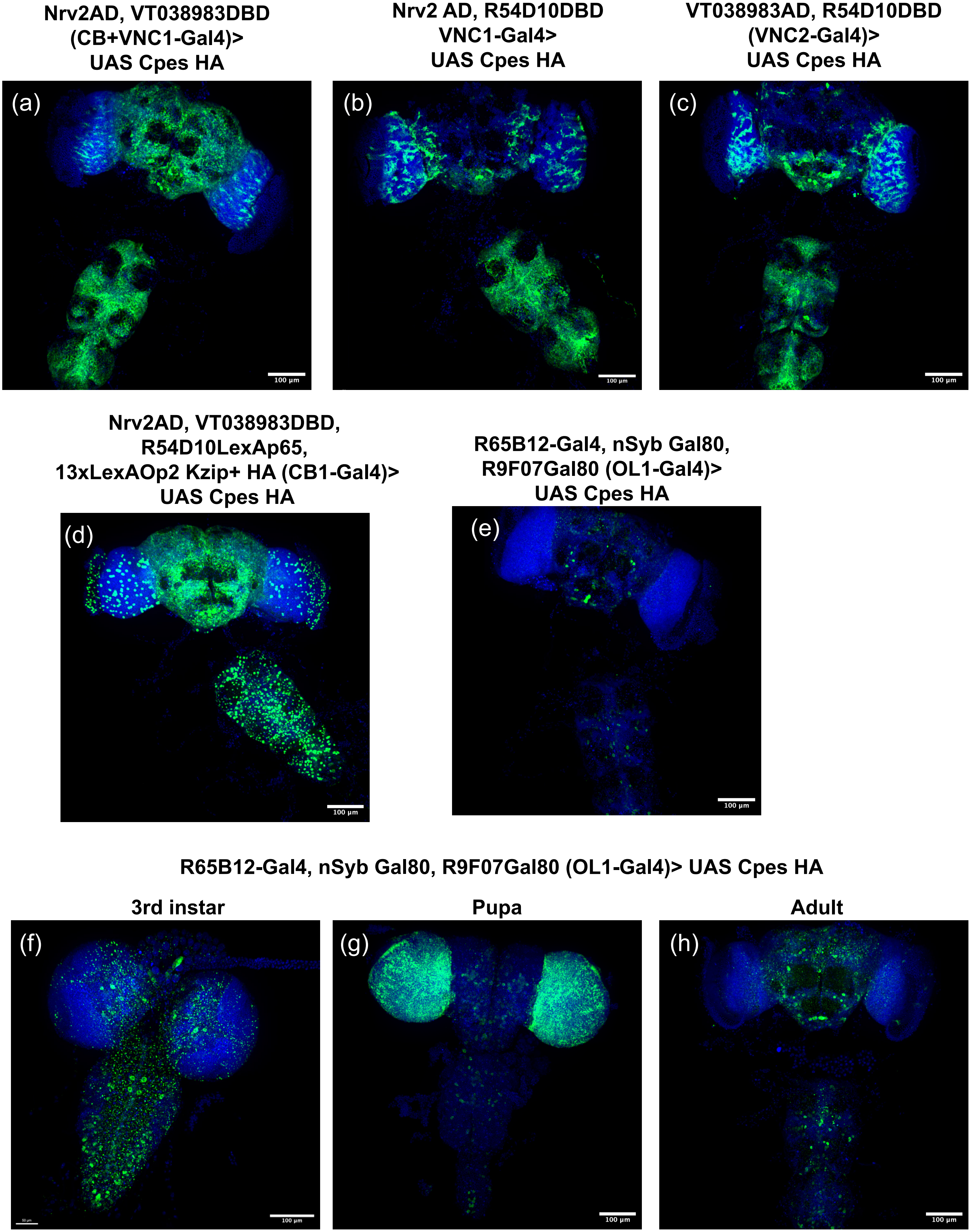
Nrv2AD, VT038983DBD (CB+VNC1-Gal4)> UAS Cpes HA
Nrv2 AD, R54D10DBD VNC1-Gal4> UAS Cpes HA
VT038983AD, R54D10DBD (VNC2-Gal4)> UAS Cpes HA
(a)
(b)
(c)
100 µm
Nrv2AD, VT038983DBD, R54D10LexAp65, 13xLexAOp2 Kzip+ HA (CB1-Gal4)> UAS Cpes HA
R65B12-Gal4, nSyb Gal80, R9F07Gal80 (OL1-Gal4)> UAS Cpes HA
(d)
(e)
R65B12-Gal4, nSyb Gal80, R9F07Gal80 (OL1-Gal4)> UAS Cpes HA
3rd instar
Pupa
Adult
(f)
(g)
(h)

Fig.S4. Localization of CPES-HA protein in cortex glial subtypes. (a) Split-Gal4 Nrv2-p65(AD), VT038983-Gal4.DBD drives the UAS-Cpes-HA expression in VNC, CB and OL of adult brain. **(b)** Split-Gal4 VT038983-p65(AD), R54D10-Gal4.DBD drives the UAS-Cpes-HA expression in VNC, SEG and OL of adult brain. **(c)** Split-Gal4 Nrv2-p65(AD), R54D10-Gal4.DBD drives the expression of UAS-Cpes-HA in VNC, SEG and OL of adult brain. **(d)** CB-specific expression of UAS-Cpes-HA using split-Gal4 Nrv2-p65(AD), VT038983-Gal4.DBD and killer zipper expression via R54D10-LexAp65(AD)>13xLexAOp2-Kzip+-HA. **(e)** OL-specific expression of UAS-Cpes-HA using R65B12 Gal4, nSybGal80 and R9F07Gal80 in adult brain and through the development **(f-h)**. Green represents HA-tagged protein, and Blue represents nuclear staining (DAPI). Scale bar in each of the image corresponds to 100μm.

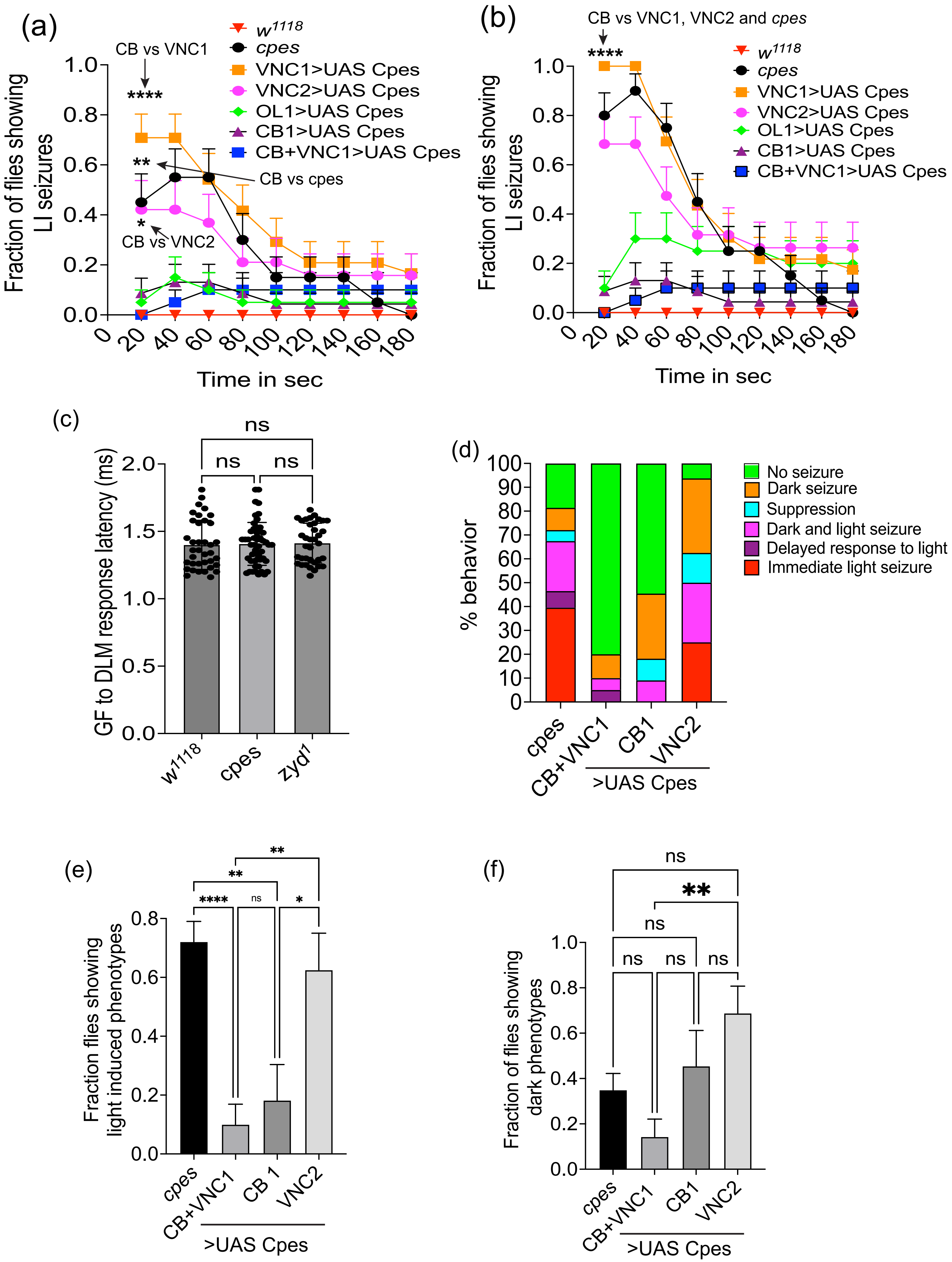

(a)
w1118
cpes
VNC1>UAS Cpes
VNC2>UAS Cpes
OL1>UAS Cpes
CB1>UAS Cpes
CB+VNC1>UAS Cpes
CB vs VNC1
****
**
CB vs cpes
*
CB vs VNC2
Fraction of flies showing LI seizures
Time in sec
(b)
CB vs VNC1, VNC2 and cpes
****
w1118
cpes
VNC1>UAS Cpes
VNC2>UAS Cpes
OL1>UAS Cpes
CB1>UAS Cpes
CB+VNC1>UAS Cpes
Fraction of flies showing LI seizures
Time in sec
(c)
ns
ns
ns
GF to DLM response latency (ms)
w1118
cpes
zyd1
(d)
No seizure
Dark seizure
Suppression
Dark and light seizure
Delayed response to light
Immediate light seizure
% behavior
cpes
CB+VNC1
CB1
VNC2
>UAS Cpes
(e)
**
**
****
ns
*
Fraction flies showing light induced phenotypes
cpes
CB+VNC1
CB 1
VNC2
>UAS Cpes
(f)
ns
**
ns
ns
ns
ns
Fraction of flies showing dark phenotypes
cpes
CB+VNC1
CB1
VNC2
>UAS Cpes

Fig.S5. Light inducible seizure analysis in single flies and quantification of electrophysiological recordings in *cpes* mutants and its rescues. (a) Flies showing complete seizures were quantified for each genotype. **(b)** flies showing complete and partial seizures were quantified for each genotype. All UAS-Cpes rescues in cortex glia are under *cpes* mutant background. Each dot and error bar at each time point represent collective behavior quantified from an average of 20 flies. Error bars in (a-b) are calculated from standard error of the mean (SEM). The 2way ANOVA multiple comparison was used to calculate P values. VNC1=Nrv2-p65(AD), R54D10-Gal4.DBD>UAS-Cpes; VNC2= VT038983-p65(AD), R54D10-Gal4.DBD>UAS Cpes; OL1=nSybGal80, R9F07Gal80, R65B12-Gal4>UAS-Cpes; CB1=Nrv2-p65(AD), VT038983-Gal4.DBD, 13xLexAOP2 Kzip+, R54D10-nlsLexAp65 > UAS-Cpes; CB+VNC1=Nrv2-p65(AD), VT038983-Gal4.DBD>UAS-Cpes. (**c**) The GF to DLM response latency was measured at room temperature and plotted as bar diagram where each dot indicates an individual fly response (time in milliseconds) that include both left and right DLMs. Statistical analysis was performed by applying ordinary one-way ANOVA, multiple comparisons. Error bars are calculated by applying standard deviation (**d**) Quantification of distinct electrophysiological responses in each genotype shown as percent behavior. (**e**) Quantification light induced phenotypes for each genotype, which included data of subtypes immediate light seizure, delayed light seizure, dark and light seizure, and suppression and shown as fraction of flies showing light induced phenotypes. (**f**) Quantification of dark/ongoing seizure for each genotype, which included data of subtypes dark and light seizure, suppression, and dark seizure alone and shown as fraction of flies showing dark phenotypes. Note, data for subtypes dark and light seizure, and suppression appear in both b and c graphs. Number of flies used for *cpes* mutant (n=43), CB+VNC1>UAS-Cpes (n=20), CB1>UAS-Cpes (n=11) and VNC2>UAS-Cpes (n=16). Statistical analysis was performed with ordinary one-way ANOVA multiple comparison and applying standard error of the mean (SEM). The P values where ****$P \leq 0.0001$; ***$P \leq 0.001$; **$P \leq 0.01$; *$P \leq 0.05$ and ns $P > 0.05$.

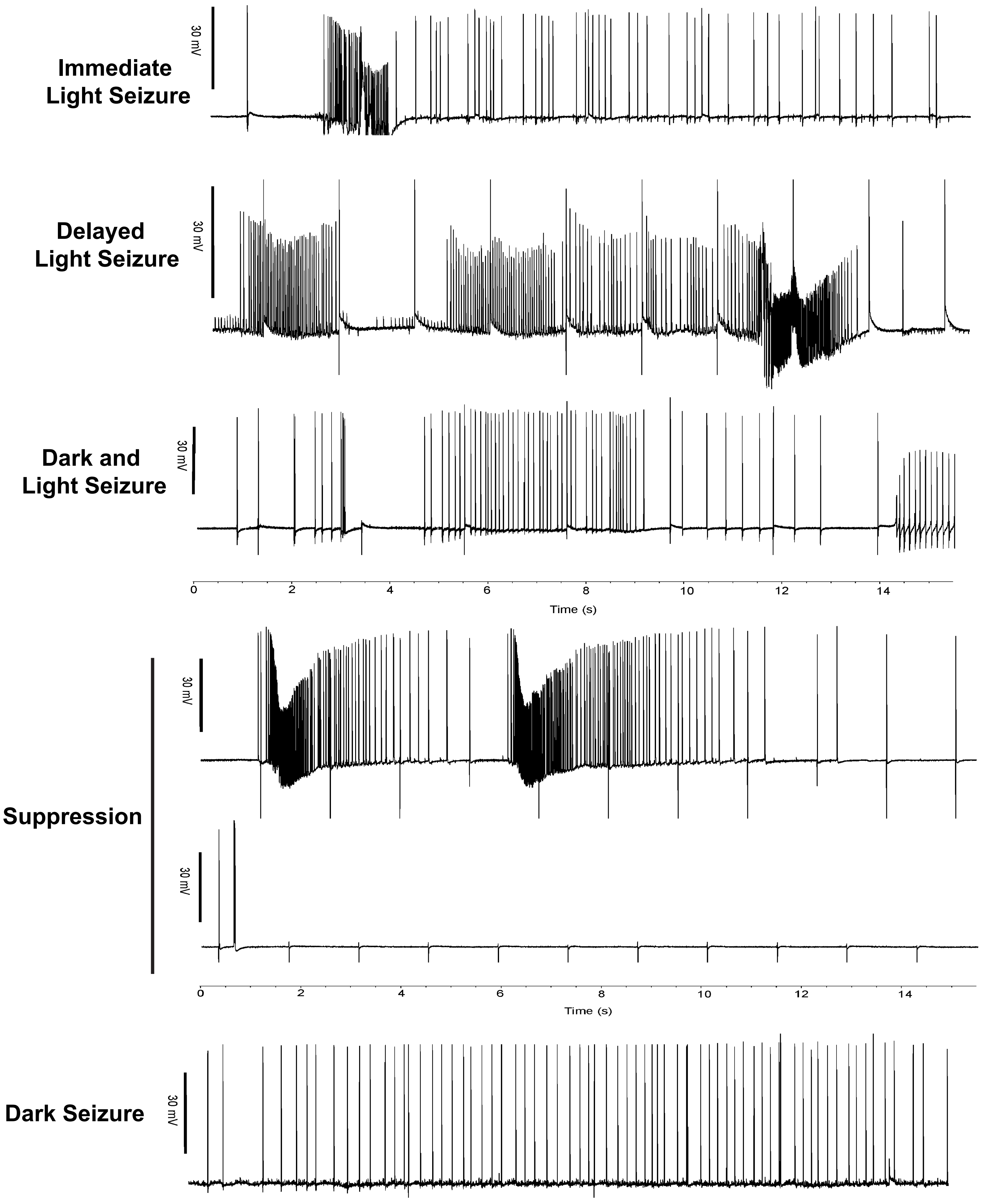

Immediate Light Seizure
30 mV
Delayed Light Seizure
30 mV
Dark and Light Seizure
30 mV
0
2
4
6
8
10
12
14
Time (s)
Suppression
30 mV
30 mV
0
2
4
6
8
10
12
14
Time (s)
Dark Seizure
30 mV

Fig.S6. Enlarged spike bursts for electrophysiological recordings in Fig.3. Boxed areas in corresponding Fig3 panels were enlarged to better represent spiking frequency and patterning. For the dark seizure trace, note that albeit continued untriggered responses were observed under light in this animal, the light did not enhance the spiking frequency observed in the dark at earlier time points or in repetitive bursts at later time points of the recording (Fig. 3). Thus, these animals were classified as seizures in dark without light induced seizures.

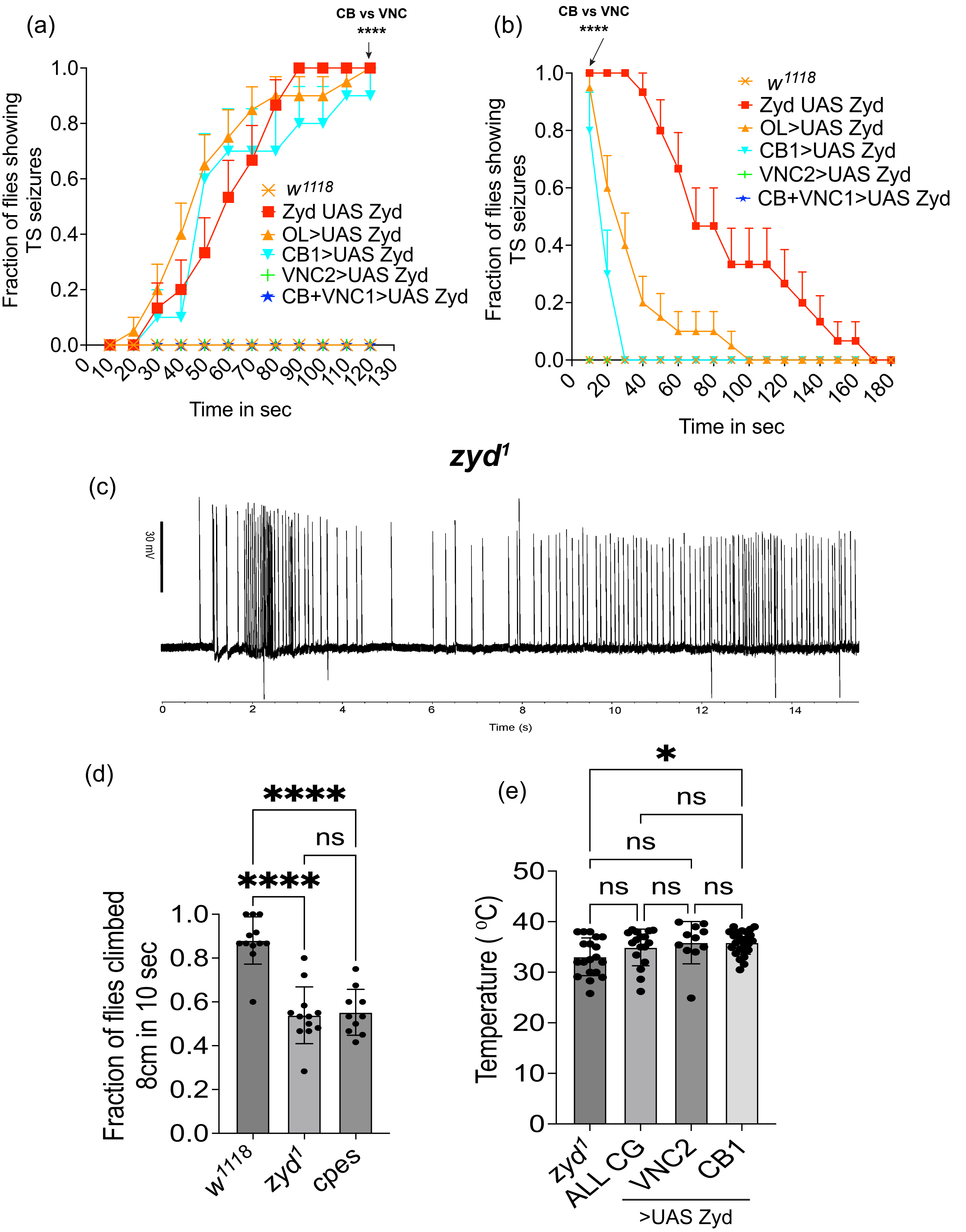

(a)
CB vs VNC
****
Fraction of flies showing TS seizures
Time in sec
w1118
Zyd UAS Zyd
OL>UAS Zyd
CB1>UAS Zyd
VNC2>UAS Zyd
CB+VNC1>UAS Zyd
(b)
CB vs VNC
****
Fraction of flies showing TS seizures
Time in sec
w1118
Zyd UAS Zyd
OL>UAS Zyd
CB1>UAS Zyd
VNC2>UAS Zyd
CB+VNC1>UAS Zyd
zyd1
(c)
30 mV
Time (s)
(d)
Fraction of flies climbed 8cm in 10 sec
****
****
ns
w1118
zyd1
cpes
(e)
*
ns
ns
ns
ns
ns
Temperature (°C)
zyd1
ALL CG
VNC2
CB1
>UAS Zyd

Fig.S7. Single-fly heat inducible seizures and electrophysiological analysis for *zyd¹* mutants. Heat inducible seizures (**a**) and recovery analysis (**b**) for single flies. Each dot and error bar in the plot indicate an average value derived collectively from 10 individual fly behaviors. Error bars in (a&b) represent standard error of the mean (SEM). Statistical analysis was performed by applying 2way ANOVA multiple comparison where ****$P \leq 0.0001$. OL=R65B12- Gal4> UAS-Zyd; CB1=Nrv2-p65(AD), VT038983-Gal4.DBD, 13xLexAOP2 Kzip+, R54D10-nlsLexAp65(AD) > UAS-Zyd; VNC2=VT038983-p65(AD), R54D10-Gal4.DBD> UAS-Zyd; CB+VNC1 =Nrv2-p65(AD), VT038983-Gal4.DBD>UAS-Zyd. (**c**) Enlarged spike burst trace for electrophysiological recordings for *zyd¹* mutants in Fig.4e. (**d**) Negative geotaxis assay in one week old wild type, *zyd¹* and *cpes* mutant flies. Each dot represents an average climbing activity of 5-10 flies in individual fly vial. Error bars represent standard deviation. (**e**) Temperature onset of seizure for *zyd¹* mutants and its rescues. Note, that temperature onset of seizures for All CG, VNC and CB rescues was calculated only from a fraction of flies that are still showing seizures between 21°C to 40°C and does not include fraction of flies that are not showing seizures. Error bars represent standard deviation in each sample. The ordinary one-way ANOVA multiple comparison was used to calculate P values where *$P \leq 0.05$ and ns $P > 0.05$ for panels d &e.

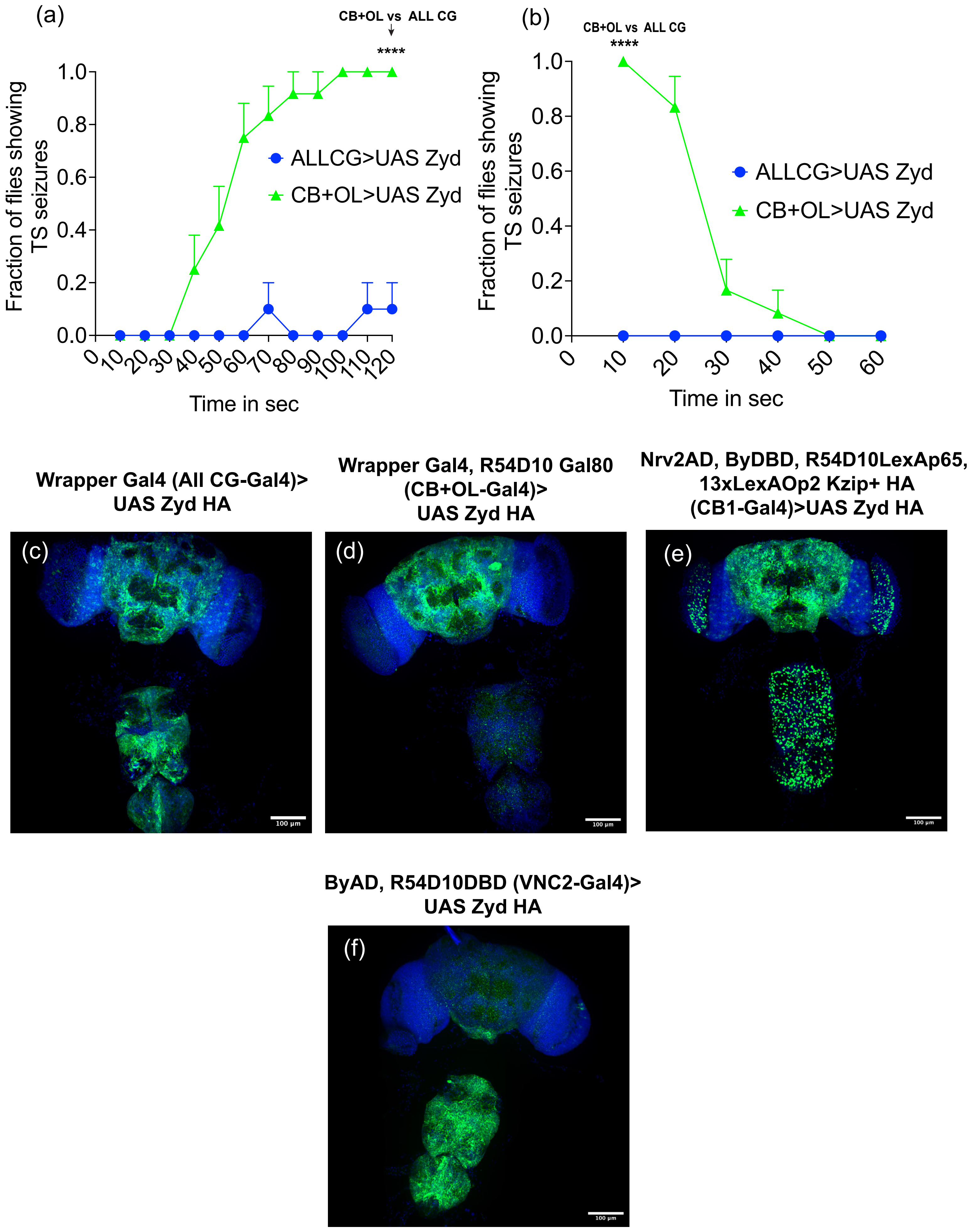
(a)
CB+OL vs ALL CG
****
Fraction of flies showing TS seizures
1.0
0.8
0.6
0.4
0.2
0.0
ALLCG>UAS Zyd
CB+OL>UAS Zyd
0
10
20
30
40
50
60
70
80
90
100
110
120
Time in sec
(b)
CB+OL vs ALL CG
****
Fraction of flies showing TS seizures
1.0
0.8
0.6
0.4
0.2
0.0
ALLCG>UAS Zyd
CB+OL>UAS Zyd
0
10
20
30
40
50
60
Time in sec
Wrapper Gal4 (All CG-Gal4)>
UAS Zyd HA
Wrapper Gal4, R54D10 Gal80
(CB+OL-Gal4)>
UAS Zyd HA
Nrv2AD, ByDBD, R54D10LexAp65,
13xLexAOp2 Kzip+ HA
(CB1-Gal4)>UAS Zyd HA
(c)
100 µm
(d)
100 µm
(e)
100 µm
ByAD, R54D10DBD (VNC2-Gal4)>
UAS Zyd HA
(f)
100 µm

Fig.S8. Single fly heat inducible seizure analysis and localization of UAS-Zyd-HA protein in cortex glial subtypes. Heat inducible seizures (**a**) and recovery analysis (**b**) for single flies. Each dot and error bar in the plot indicate an average value derived collectively from 10 individual fly behaviors. Error bars in (a&b) represent standard error of the mean (SEM). Statistical analysis was performed by 2way ANOVA, multiple comparison, where ****P ≤ 0.0001. All CG =R54H02-Gal4>UAS-Zyd; CB+OL=R54H02-Gal4, R54D10- Gal80>UAS-Zyd. **(c-f)** Adult fly brains expressing HA-tagged Zyd protein in cortex glia. (**c**) Wrapper-Gal4 drives expression of UAS-Zyd-HA in all cortex glia. (**d**) Wrapper-Gal4, R54D10-Gal80 drives UAS-Zyd-HA expression strongly in CB. (**e**) Nrv2-p65(AD), VT038983-Gal4.DBD, R54D10-LexAp65,13xLexAOp2 Kzip+-HA drives UAS-Zyd-HA expression strongly in CB. Punctate expression in VNC and OL indicates expression of HA-tagged killer zipper. (**f**) VT038983-p65(AD) and R54D10-Gal4.DBD drives UAS-Zyd-HA expression strongly in VNC and SEG. Green represent immunostaining for HA-tag, blue represent nuclear staining with DAPI. Scale bar in each of the image corresponds to 100μm.

R65B12Gal4, R9F07 Gal80 >UAS Zyd HA

3rd instar | **Pupa** | **Adult**

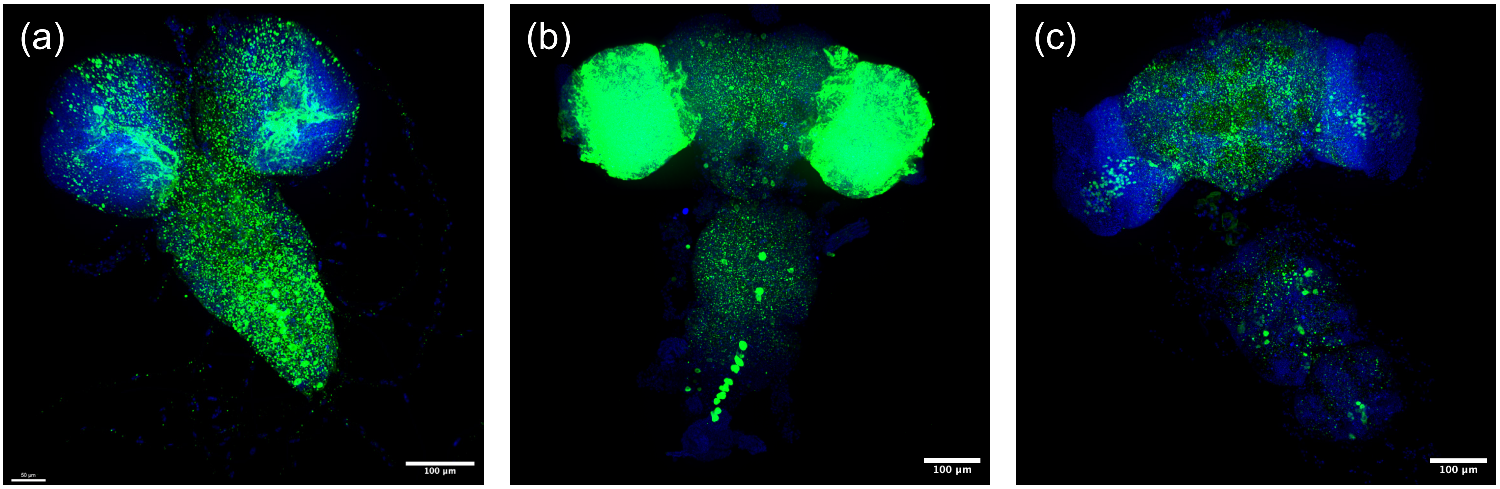

R65B12Gal4 >UAS Zyd HA

3rd instar | **Pupa** | **Adult**

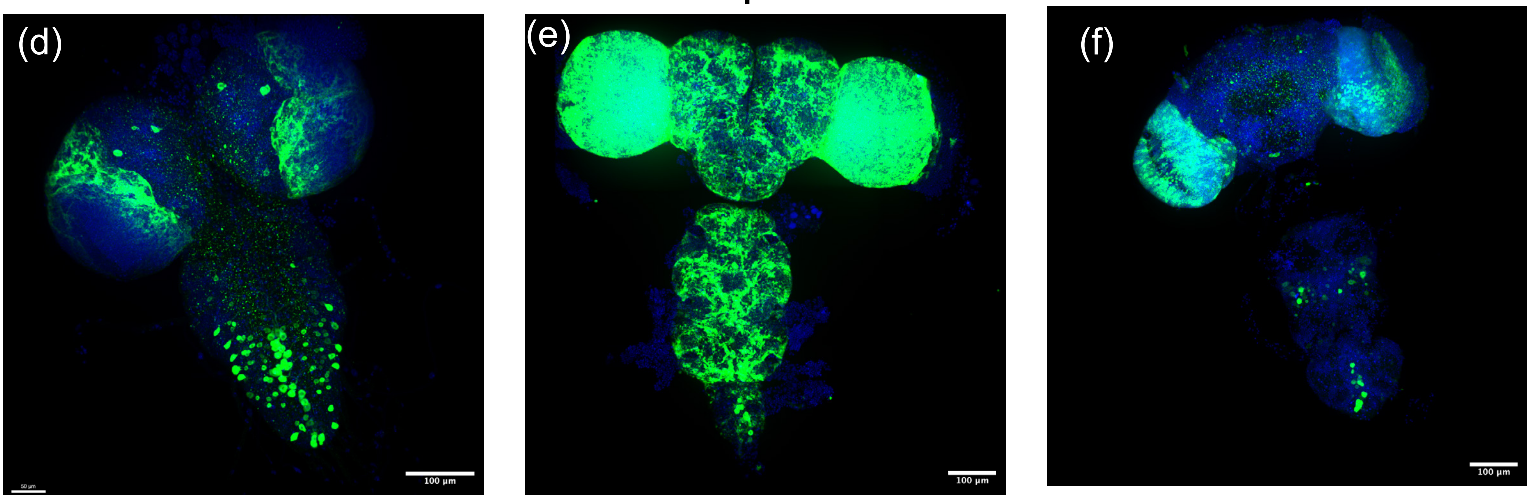

Fig.S9. Immunostaining analysis for OL specific UAS-Zyd-HA expression in the presence or absence of R9F07 Gal80. (**a-c**) R65B12Gal4 expression in the presence of R9F07 Gal80. (**d-f**) R65B12 Gal4 expression in the absence of R9F07Gal80. ZYD HA protein (Green), cell nuclei (blue). Scale bar corresponds to 100μm.

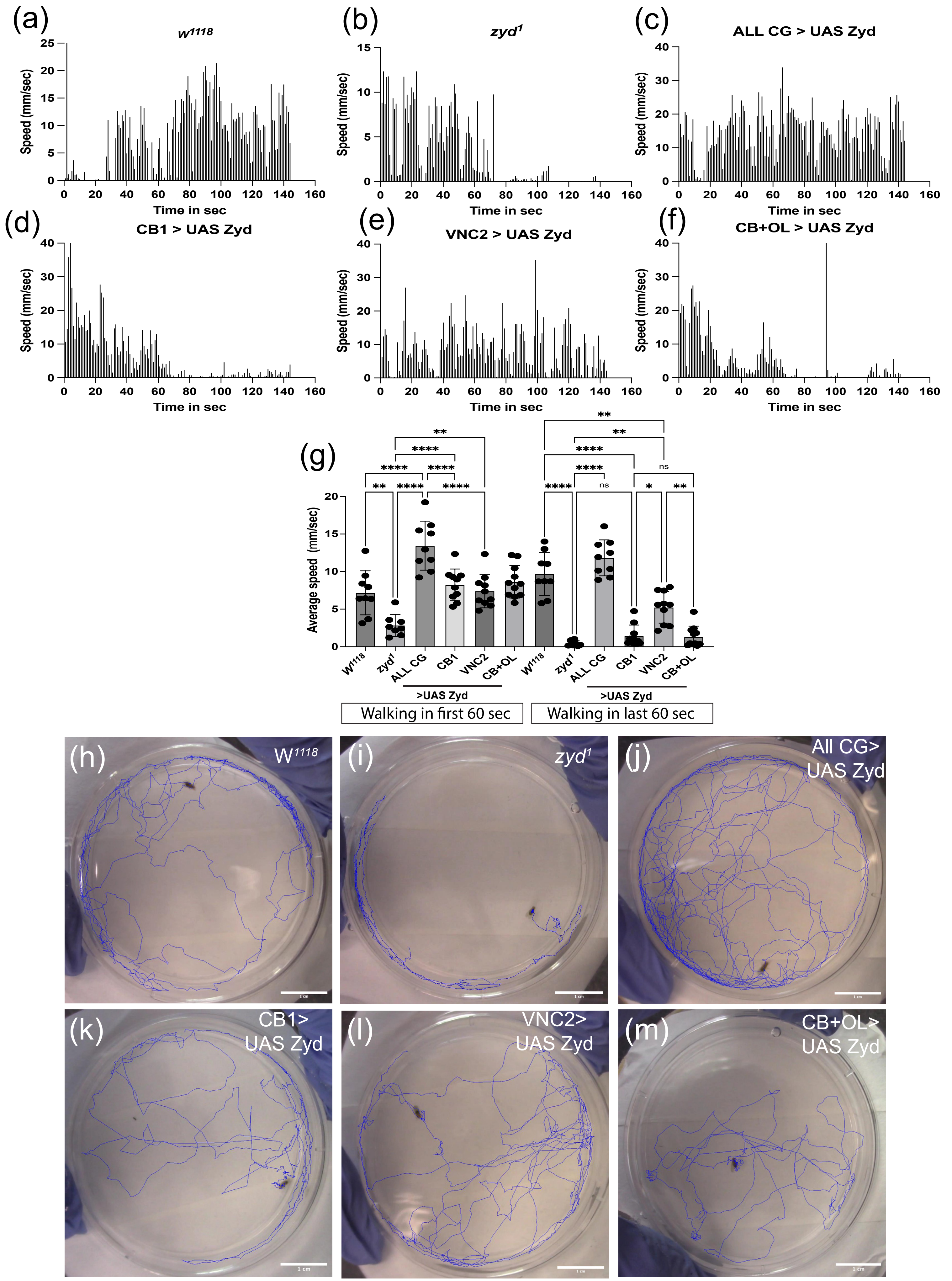

(a)
w^{1118}
Speed (mm/sec)
Time in sec
(b)
zyd^1
Speed (mm/sec)
Time in sec
(c)
ALL CG > UAS Zyd
Speed (mm/sec)
Time in sec
(d)
CB1 > UAS Zyd
Speed (mm/sec)
Time in sec
(e)
VNC2 > UAS Zyd
Speed (mm/sec)
Time in sec
(f)
CB+OL > UAS Zyd
Speed (mm/sec)
Time in sec
(g)
Average speed (mm/sec)
W^{1118}
zyd^1
ALL CG
CB1
VNC2
CB+OL
>UAS Zyd
Walking in first 60 sec
Walking in last 60 sec
ns
(h)
W^{1118}
(i)
zyd^1
(j)
All CG>
UAS Zyd
(k)
CB1>
UAS Zyd
(l)
VNC2>
UAS Zyd
(m)
CB+OL>
UAS Zyd

Fig.S10. Tracking of single-fly walking and heat inducible seizures in single flies. (a-f) Single-fly waking was traced for the entire duration (2 min 25 sec) of the assay at 38.5 °C in *zyd*^{1} mutants and its rescues. **(g)** Distance walked in first 60 seconds and last 60 seconds was averaged and plotted. Each dot in g represents a single-fly walking distance per second (n=10). The 2way ANOVA multiple comparison was used to calculate P values where ****P ≤ 0.0001; ***P ≤ 0.001; **P ≤ 0.01; *P ≤ 0.05 and ns P >0.05. **(h-m)** Single fly waking was traced for the entire duration (2 min 25 sec) of the assay. All CG=R54H02-Gal4>UAS-Zyd; CB=Nrv2-p65(AD), VT038983-Gal4.DBD, 13xLexAOP2 Kzip+, R54D10-nlsLexAp65AD>UAS-Zyd; VNC2=VT038983-p65(AD), R54D10-Gal4.DBD>UAS-Zyd; CB+OL=R54H02-Gal4, R54D10-Gal80>UAS-Zyd.

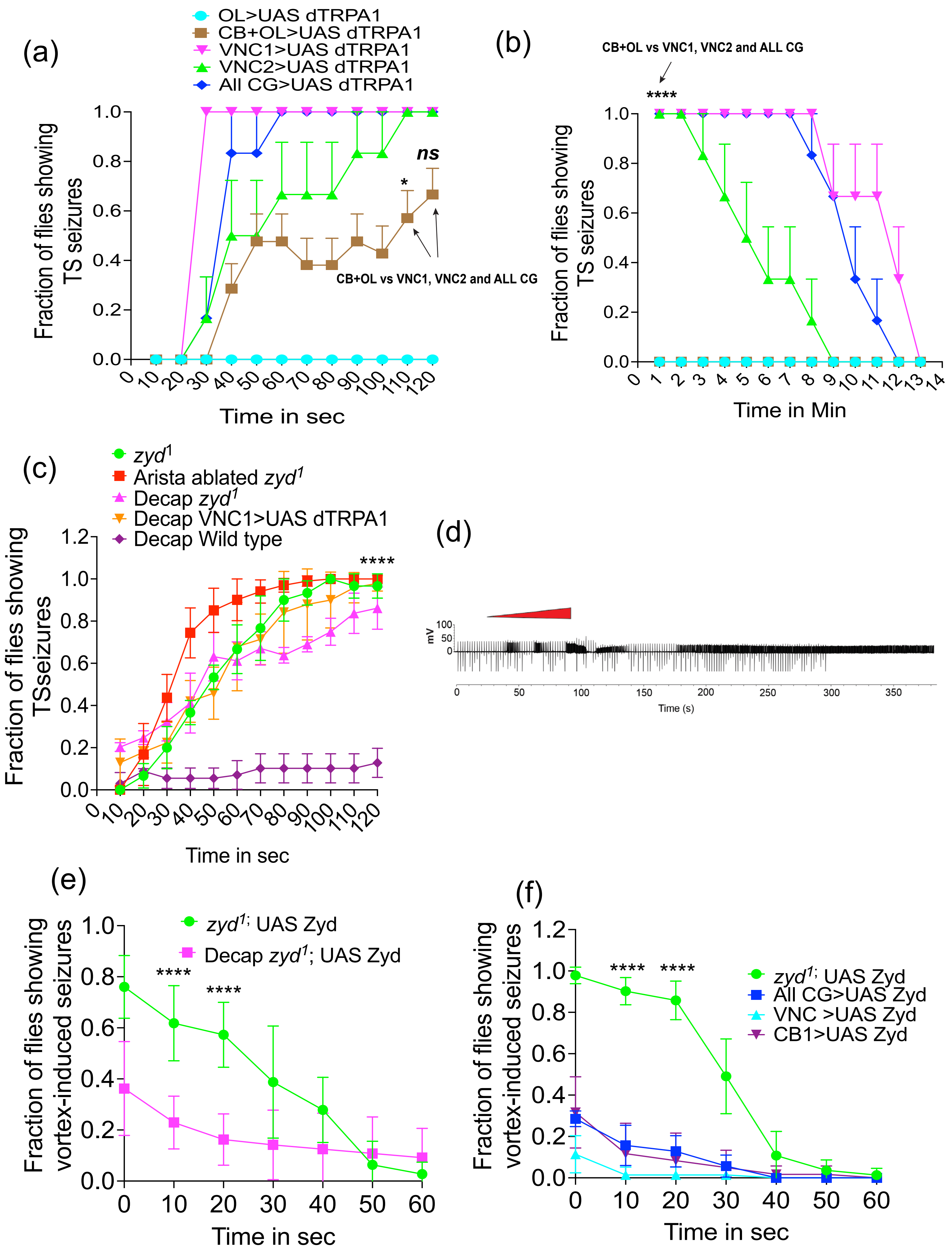
(a)
OL>UAS dTRPA1
CB+OL>UAS dTRPA1
VNC1>UAS dTRPA1
VNC2>UAS dTRPA1
All CG>UAS dTRPA1
Fraction of flies showing TS seizures
Time in sec
ns
*
CB+OL vs VNC1, VNC2 and ALL CG
(b)
CB+OL vs VNC1, VNC2 and ALL CG
****
Fraction of flies showing TS seizures
Time in Min
(c)
zyd1
Arista ablated zyd1
Decap zyd1
Decap VNC1>UAS dTRPA1
Decap Wild type
****
Fraction of flies showing TSseizures
Time in sec
(d)
mV
Time (s)
(e)
zyd1; UAS Zyd
Decap zyd1; UAS Zyd
****
****
Fraction of flies showing vortex-induced seizures
Time in sec
(f)
zyd1; UAS Zyd
All CG>UAS Zyd
VNC >UAS Zyd
CB1>UAS Zyd
****
****
Fraction of flies showing vortex-induced seizures
Time in sec

Fig.S11. Central brain function is important for vortex induced seizures but not for heat inducible seizures. (a) Measurement of heat inducible seizures in single flies overexpressing UAS-dTRPA1 in cortex glia. **(b)** Recovery analysis for the single fly data in (a). Error bars in (a-b) are calculated from standard error of the mean (SEM). All CG=R54H02-Gal4>UAS-dTRPA1; CB+OL= R54H02-Gal4, R54D10-Gal80>UAS-dTRPA1; OL=nsybGal80, R65B12-Gal4>UAS-dTRPA1; VNC1= Nrv2-p65(AD), R54D10-Gal4 DBD> UAS-dTRPA1; VNC2= VT038983-p65(AD), R54D10-Gal4.DBD>UAS-dTRPA1. **(c)** Temperature sensitive assay with arista ablated, and decapitated adult flies. Each data point in (c) represents average behavior of about 50 flies from 3 independent experiments. **(d)** Electrophysiological responses of DLMs of a decapitated zyd^1 mutant when exposed to increased temperature (red triangle). **(e)** vortex/mechanical induced seizures in intact or decapitated zyd^1 flies. Each dot represents an average behavior of 50-100 flies. **(f)** vortex induced seizures in zyd^1 mutants and its cortex glial subtype specific rescues. Each dot represents an average behavior from at least 30 flies.

Table S1: List of Gal4 Drivers from FlyLight project at Janelia whose expression detected in cortex glia of adult brain.

VT002963-Trissin receptor
VT010664-skywalker
VT026012-Ionotrophic receptor64a
VT038983-blistry (by) tensin interacts with integrins.
R9F07-retn
R10D01-bifid (T-box transcription factor)
R14C04-nrv2 (non-catalytic component of Na+ K+
R15A06-similar (sima-transcriptional regulator of hypoxia)
R15B11-Ork1 Open rectifier K+ channel1
R17F05-acyl-CoA synthase long chain (AcsI)
R20B06-nAChRalpha3
R24F07-anachronsm (ana secreted glycoprotein expressed in glia)
R30E04- (ocelliless) transcriptional regulator, regulate rhodopsin expression.
R31F07-string, tyrosine protein phosphatase
R33A09-SNF4/AMP activated protein kinase gamma subunit-lipid metabolism.
R40B10-chinmo-BTB-zinc finger transcription factor
R47A02-Liprin gamma
R54H02-wrapper
R64C02-Na pump a subunit
R64C07 Na pump a subunit
R73F09-octa2R-a2-adrenergic like octopamine receptor
R77A03-MFS9-Major facilitator superfamily transporter 9
R81G12-NFAT-NFAT nuclear factor
R91G05-sidestepVIII-expressed in RP neurons and adult head and pCC neurons

Table S2: List of Gal4/Split-Gal4/LexA/Gal80/Kzip combinations used in this study for brain region specific cortex glial expression.

**Drivers**					
Brain region	Genotype	3rd instar	pupa	adult	Comment
**CB+VNC1** (Fig.1a-c)	Nrv2-p65(AD), VT038983-Gal4(DBD).	CB+VNC	CB+VNC	CB+VNC+OL	Additional expression in adult OLs
**CB+VNC2** (Fig.S1a-c)	R9F07-p65(AD), VT038983-Gal4(DBD)	CB+VNC	CB+VNC	Mostly CB+VNC	Weak expression in one of the two OLs
**OL** (Fig.S1d-f)	R65B12-Gal4	OL	OL+CB+VNC	OL	Expression in all cortex glia in pupal stage
**OL1** (Fig.1d-f)	R65B12-Gal4, R9F07-Gal80, nSyb-Gal80	OL	OL	OL	Restricted expression to OLs in all stages
**VNC1** (Fig.S2a-c)	Nrv2-p65(AD), R54D10DBD	VNC	VNC	VNC+SEG+OL	Additional expression in OLs and SEG in adult brain
**VNC2** (Fig.1g-i)	VT038983 (AD) R54D10.DBD	VNC+CB*	VNC	VNC+SEG+OL	*Additional expression in 3rd instar central brain neuroblast lineages. In the adult brain expression also occurs in SEG and OL
**CB1** (Fig.1j-l)	Nrv2-p65(AD), VT038983-Gal4(DBD), R54D10-(LexA), 13xLexAop2 Kzip+.	CB*	CB*	CB*	*Tip of the VNC continues to show expression in 3rd instar and pupa but weakly in adult.
**CB2** (Fig.S3g-i)	Nrv2-p65 (AD), Wrapper Gal4 (DBD), R54D10-(LexA), 13xLexAop2 Kzip+.	CB*	CB*	CB*	*Tip of the VNC continues to show expression in 3rd instar and pupa but weakly in adult.
**All cortex glia (Ctx-Gal4)** Fig.S3a-c	Nrv2-p65 (AD), Wrapper Gal4 (DBD),	All cortex glia	*All cortex glia	All cortex glia	*Expression is weaker in optic lobes
**All cortex glia**	R54H02-nlsLexAp65AD,	NA	NA	All cortex glia	Expresses in all cortex glia of the adult CNS.

(Fig.2a & b)	LexAop2-rCD2-GFP				
**CB+VNC1 CPES rescue** (Fig.2c)	R54H02-nlsLexAp65AD, LexAop2-rCD2-GFP, Nrv2-p65(AD) and pVT038983-Gal4.DBD, UAS CPES	NA	NA	Full rescue in VNC+CB but partial rescue in OL	Full rescue in VNC+CB but partial rescue in OL
**VNC2 CPES rescue** (Fig.2d)	R54H02-nlsLexAp65, LexAop2-rCD2-GFP, VT038983-p65(AD), and R54D10-Gal4.DBD, UAS CPES	NA	NA	Full rescue in VNC and SEG. No rescue in CB and OLs	Full rescue in adult VNC and SEG. No rescue in CB and OLs.
**OL1 CPES rescue** (Fig.2e)	R54H02-nlsLexAp65, LexAop2-rCD2-GFP, R65B12-Gal4, R9F07Gal80	NA	NA	Full rescue in OLs and partial rescue in CB.	Full rescue in OLs and partial rescue in CB. No rescue in VNC.
**All cortex glia** (Fig.5a-c)	R54H02-Gal4 (Wrapper Gal4)	All cortex glia	All cortex glia	All cortex glia	All cortex glia
**CB+OL** (Fig.5d-f)	R54H02-Gal4, R54D10 Gal80	CB+OL	CB+OL	CB and weakly in OL	Repression is incomplete in adult OLs.

Table.S3: List of primers used for cloning in this study.

R54D10 FW	ggggACAAgTTTgTACAAAAAAgCAggCTTCgTCTACATCgACAAggCATCCgAgT
R54D10 Rev	ggggACCACTTTgTACAAgAAAgCTgggTCgTTTgCggTgCgATCgCCATTTTTg
R9F07 FW	ggggACAAgTTTgTACAAAAAAgCAggCTTCCACCCAgggTgATCCAATTCgC
R9F07 Rev	ggggACCACTTTgTACAAgAAAgCTgggTCTCAgTgCCAAATgCCAAA
Zyd FW	ggggACAAgTTTgTACAAAAAAgCAggCTTCACCATgAAATATATAAATTgCACTCAgCCCgCC
Zyd Rev	ggggACCACTTTgTACAAgAAAgCTgggTCTgATCgACCgCAggTgggCAggTTgAC
Cpes FW	ggggACAAgTTTgTACAAAAAAgCAggCTTCACCATgATCggACCCAgTTCgCAgATC
Cpes Rev	ggggACCACTTTgTACAAgAAAgCTgggTCCAgTgAAATATTACTgCACgCCgTAgAg

Data S1

	Assay 1	Assay 2	Assay 3
1	c	c	c
2	p	p	c
3	c	p	p
4	no	no	p
5	p	no	c
6	c	no	c
7	no	no	no
8	no	no	no
9	no	no	no
10	p	c	c
11	c	p	p
12	p	c	c
13	no	no	no
14	p	p	p
15	no	c	no
16	p	c	p
17	no	no	p
18	c	c	c
19	no	no	c
20	c	no	no
21	p	c	p
22	no	no	no
23	no	c	c
24	c	c	no
25	c	c	c
26	p	p	c
27	no	c	p
28	c	c	p
29	p	c	p

Data S2

time	Fly1	Fly2	Fly3	Fly4	Fly5	Fly6	Fly7	Fly8	Fly9	Fly10	Fly11	Fly12	Fly13	Fly14	Fly15	Fly16	Fly17	Fly18	Fly19	Fly20	Fly21
10	0	0	0	0	0	0	0	0	0	0	0	0	0	0	0	0	0	0	0	0	0
20	0	0	0	0	0	0	0	0	0	0	0	0	0	0	0	0	0	0	0	0	0
30	0	0	0	0	0	0	0	0	0	0	0	0	0	0	0	0	0	0	0	0	0
40	0	0	1	1	1	0	1	0	0	0	0	0	0	0	0	1	0	0	0	0	1
50	1	0	1	1	1	0	1	0	0	1	1	0	1	0	0	1	0	0	1	0	0
60	1	0	0	1	1	0	1	0	1	1	1	0	1	0	0	1	0	0	1	0	0
70	1	0	0	1	1	0	0	0	1	1	1	0	1	0	0	0	0	0	1	0	0
80	1	0	0	1	1	0	0	0	1	1	0	1	1	0	0	0	0	0	1	0	0
90	1	0	0	1	1	1	1	0	1	0	1	1	1	0	0	0	0	0	1	0	0
100	1	1	0	1	1	0	0	0	1	0	1	1	1	0	0	0	0	0	1	0	0
110	1	1	0	1	1	0	1	0	1	1	1	1	1	0	1	0	0	0	1	0	0
120	1	1	0	1	1	0	1	1	1	1	1	1	1	1	1	0	0	0	1	0	0

0= fly is not in seizure, i.e standing

1=fly is in seizure, i.e., on its back of sides

References

1. G. Kunduri *et al.*, Defective cortex glia plasma membrane structure underlies light-induced epilepsy in cpes mutants. *Proc Natl Acad Sci U S A* **115**, E8919–E8928 (2018).
2. J. C. Coutinho-Budd, A. E. Sheehan, M. R. Freeman, The secreted neurotrophin Spatzle 3 promotes glial morphogenesis and supports neuronal survival and function. *Genes Dev* **31**, 2023–2038 (2017).
3. J. E. Melom, J. T. Littleton, Mutation of a NCKX eliminates glial microdomain calcium oscillations and enhances seizure susceptibility. *J Neurosci* **33**, 1169–1178 (2013).
4. W. R. Williamson, P. R. Hiesinger, Preparation of developing and adult Drosophila brains and retinae for live imaging. *J Vis Exp* 10.3791/1936 (2010).
5. S. Weiss, J. E. Melom, K. G. Ormerod, Y. V. Zhang, J. T. Littleton, Glial Ca(2+)signaling links endocytosis to K(+) buffering around neuronal somas to regulate excitability. *Elife* **8** (2019).
6. M. J. Allen, T. A. Godenschwege, Electrophysiological recordings from the Drosophila giant fiber system (GFS). *Cold Spring Harb Protoc* **2010**, pdb prot5453 (2010).
7. J. R. Kroll, K. G. Wong, F. M. Siddiqui, M. A. Tanouye, Disruption of Endocytosis with the Dynamin Mutant shibirets1 Suppresses Seizures in Drosophila. *Genetics* **201**, 1087–1102 (2015).
8. D. Kuebler, M. A. Tanouye, Modifications of seizure susceptibility in Drosophila. *J Neurophysiol* **83**, 998–1009 (2000).
9. J. Lee, C. F. Wu, Electroconvulsive seizure behavior in Drosophila: analysis of the physiological repertoire underlying a stereotyped action pattern in bang-sensitive mutants. *J Neurosci* **22**, 11065–11079 (2002).
10. Y. O. Ali, W. Escala, K. Ruan, R. G. Zhai, Assaying locomotor, learning, and memory deficits in Drosophila models of neurodegeneration. *J Vis Exp* 10.3791/2504 (2011).
